# An Overview of the Potential Therapeutic Applications of CO-Releasing Molecules

**DOI:** 10.1155/2018/8547364

**Published:** 2018-08-12

**Authors:** Aiten Ismailova, David Kuter, D. Scott Bohle, Ian S. Butler

**Affiliations:** Department of Chemistry, McGill University, 801 Sherbrooke Street West, Montreal, QC, Canada H3A 3K6

## Abstract

Carbon monoxide (CO) has long been known as the “silent killer” owing to its ability to form carboxyhemoglobin—the main cause of CO poisoning in humans. Its role as an endogenous neurotransmitter, however, was suggested in the early 1990s. Since then, the biological activity of CO has been widely examined via both the direct administration of CO and in the form of so-called “carbon monoxide releasing molecules (CORMs).” This overview will explore the general physiological effects and potential therapeutic applications of CO when delivered in the form of CORMs.

## 1. Introduction

Carbon monoxide (CO) is a colorless, odorless gas that is endogenously produced in the human body through heme oxygenase, which is present in two forms: the constitutive (HO-2 and HO-3) and inducible (HO-1) isoforms [1]. It has a higher affinity for hemoglobin than does O_2_, when it forms carboxyhemoglobin, thus preventing O_2_ transport throughout the body. A few years ago, it was discovered that CO gas in small doses exhibits some anti-inflammatory and antimicrobial properties making it useful as a potential therapeutic agent for disease control [2]. Unfortunately, using CO gas under clinical conditions is not practical. The gas has a low solubility in water, and so it has only a limited solubility in body fluids, which means that a person would need to inhale a high concentration of the gas to attain a meaningful concentration in the body [3]. Moreover, the delivery of gaseous CO cannot be precisely controlled and overexposure of body tissue to the gas could be harmful [4].

It is the well-known synergistic bonding (*σ*-donor and *π*-acceptor) ability of CO to transition metals that accounts for both its stability and reactivity [5]. Countless transition metal carbonyl complexes are now known, and some of these can release CO within the human body without affecting the level of carboxyhemoglobin produced. These complexes are now referred to as “carbon monoxide releasing molecules (CORMs).” A CORM is made up of two parts: a *CORM sphere* and a *drug sphere*. The CORM sphere determines the number of CO molecules that can be released, the kinetics of the CO release, and the trigger mechanism necessary to cause the CO release. The drug sphere, defined by the periphery of the coligands surrounding the transition metal center, affords the most critical advantage of a CORM over CO gas. This advantage is because the CORM can modulate the partition ratio between the various body fluids and tissues, thus allowing it to be targeted to specific tissues. Most of the recent research on CORMs has been focused on the CO-release properties, and the drug sphere has been essentially ignored. Since the drug sphere is essential for targeting the CORM into a desired area of the body, more research is needed in this area [6].

**Scheme 1:**
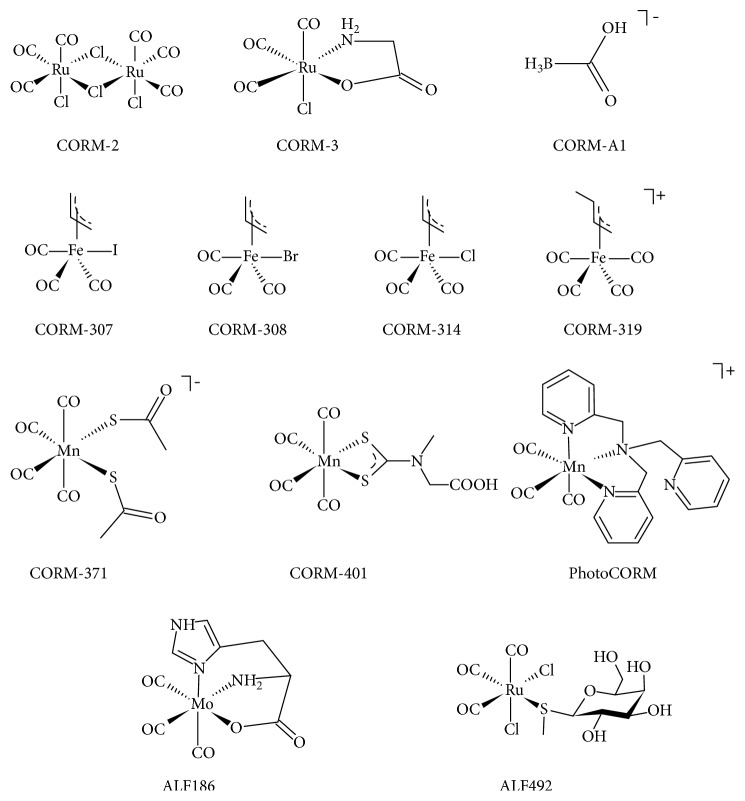


The Ph.D. thesis of Joao Daniel de Silva Seixas from the Institute of Chemistry and Biology at the New University of Lisbon in 2011 entitled *Development of CO-Releasing Molecules for the Treatment of Inflammatory Diseases* is particularly informative about the utility of CORMs in some medical situations [7]. Interestingly, one of the first CORM molecules ever identified was Et_4_N[Mo(CO)_5_Br] (ALF062), and this was prepared during the Ph.D. thesis work of one of the authors of this overview during the early 1960s [8]. Several important review articles on CORMs have been published over the years [9–14]. The present overview is focused on the pharmacological uses of CORMs and their impact on various human pathologies with the aim of determining whether CORMs will indeed be useful as therapeutic agents as was originally suggested in the pioneering paper by Motterlini in 2002 [1]. Reviews somewhat related to ours have recently been published by Ward [15] and Ling et al. [16]. The structures of the CORMs mentioned here are shown in Scheme 1, and a summary of the potential therapeutic applications is listed in Table 1.

## 2. Effect of CORMs on Bacteria

CORMs possess the ability to accumulate inside bacterial cells before they release CO, and this fact has led them to become useful CO donors to bacterial targets, such as *Escherichia coli*, *Staphylococcus aureus*, *Helicobacter pylori,* and *Pseudomonas aeruginosa*. For instance, Bang et al. [17] have demonstrated that dimeric CORM-2, Ru_2_Cl_4_(CO)_6_, decreases the bacterial viability of multidrug resistant uropathogenic isolates of *E. coli* (UPEC) under biofilm conditions as well as in the colonization of human bladder epithelial cells. Biofilm is the term given to bacteria enclosed in a matrix that adheres to an abiotic or a biotic surface. There is increased resistance to antimicrobial agents by many bacteria when grown in biofilms. The formation of biofilm on medical devices, such as urinary catheters, increases the likelihood of urinary tract infections (UTIs). In patients with UTI, UPEC, and biofilm-producing UPEC are frequently seen. An example of a bacterium that causes biofilm formation is extended-spectrum beta-lactamase- (ESBL-) producing *Enterobacteriaceae*. The objective of this 2016 study of Bang et al. [17] was to investigate the antibacterial effects of CORM-2 on ESBL-producing UPEC in biofilm and in the colonization of human bladder epithelial cells. The results revealed that CORM-2 exhibited antibacterial properties against drug-resistant UPEC both under biofilm and host cell colonization conditions. CORM-2 reduced planktonic bacterial counts in the exponential growth phase by more than 3 log units within 4 hours. Although CORM-2 showed a delayed inhibitory response in the stationary growth phase, an antibacterial effect was observed after 24 hours. To confirm that CO is necessary for the antibacterial effect of CORM-2, the CO-free molecule, Ru(DMSO)_4_Cl_2_, was exposed to ESBL biofilms and epithelial cells, and unlike CORM-2, it demonstrated no reduction.

Another study on the efficacy of CORMs against *E. coli* has been reported by Tinajero-Trejo et al. [18]. Instead of using CORM-2, however, they employed the novel water-soluble, Mn-based photoactivated CORM, Mn(CO)_3_(tpa-k^3^*N*), known as PhotoCORM. Upon irradiation at 325 nm, the complex releases CO. Tested specifically against the EC958 strain of *E. coli*, it was shown that the illuminated PhotoCORM suppressed respiration of EC958 membranes when compared to the untreated samples at a 50% O_2_ tension and even more so at a much lower O_2_ tension (15%). The PhotoCORM also inhibited EC958 growth on glucose upon illumination, but not in the dark. Although the antimicrobial effects of PhotoCORM depend only on light activation, it is independent of O_2_, suggesting that PhotoCORM is toxic in anoxic cultures as well. A related photoactivated CORM, Trypto-CORM, also displays marked cytotoxicity toward *E. coli* and, more recently, has been shown to be effective against *Neisseria gonorrhoeae* [19, 20].

In addition to *E. coli,* the effect of CORMs against the leading pathogen in skin infections, *Staphylococcus aureus*, has been explored. This bacterium can form biofilms that are almost impossible to eradicate using regular antibiotics. The goal of a study by Klinger-Strobel et al. [89] was to test the ability of CO to eliminate this highly resistant bacterium. The CO-treated biofilms showed elevated counts of dead bacteria compared to other biofilms that were not exposed to CO gas. Bacterial cells within methicillin-resistant *S. aureus* biofilms, the most common pathogen that causes skin infections, were killed after 135 min exposure to 405 nm light in the presence of CO gas. Furthermore, based on the work of Tavares et al. [21], CORMs prevented the survival of *Helicobacter pylori*, a pathogen responsible for chronic gastric ulcers. Treatment with CORM-2, which is soluble in DMSO, for 15 hours resulted in a 4-log loss of cell viability; however, when treated with water-soluble CORM-3, Ru(CO)_3_Cl(glycinate), the viability was attenuated by 2-log only. Thus, a more potent effect against *H. pylori* is achieved by CORM-2. This difference is presumably due to the different reactivity, hydrophobicity, polarity, and/or hydrogen bonding ability. Specifically, the DMSO-soluble CORM-2 is more hydrophobic than is the water-soluble CORM-3, which may favour the interaction of CORM-2 with the medium and the *H. pylori* cells. Additionally, exposure to CORM-2 for 5 min caused a decrease of greater than 50% in O_2_; this suggests that CORM-2 inhibits *H. pylori* cell respiration, thereby inducing bacterial death. The mechanism for bacterial extermination ability of CORM-2 can be traced to the ability of *H. pylori* to express a nickel-containing urease, an enzyme responsible for the capability of the bacteria to cause disease. Since CO can bind transition metals, the effect of CORM-2 on the urease activity was analyzed. The results indicated that *H. pylori* grown in the presence of CORM-2 demonstrated a 65% decrease in urease activity. Furthermore, when the bacterial cells were incubated with increasing concentrations of CORM-2 for 15 min, the urease activity was completely abolished. Thus, the antibacterial effect induced by CORM-2 is most probably due to the ability of CO to bind to nickel.

The enteropathogen, *S. typhimurium*, which is associated with gastroenteritis, survives and proliferates within innate immune cells. Research conducted by Rana et al.co [22] has assessed the effects of CORMs on the bacterium, *Salmonella enterica serova typhimurium.* With an increasing concentration of CORM-3, both the growth and viability of *S. typhimurium* were reduced. The CO released from CORM-3 binds to the terminal oxidases of *S. typhimurium* and is rapidly taken up by the *Salmonella* cells. The ruthenium complex, CORM-3, speedily fluxes into the *Salmonella* cells. After 80 min, the level of ruthenium inside the cells exceeded the final concentration added to the extracellular medium, indicating that CORM-3 is actively concentrated into bacterial cells.

Desmard et al. [23] have demonstrated that different antibacterial activities occur against *Pseudomonas aeruginosa* depending on the structural nature of the CORM used. Both CORM-2 and CORM-3 contain ruthenium and are rapid CO liberators—CO release occurs within 1 min after their addition to biological systems. These two compounds only differ in their solubility in DMSO for CORM-2 and in water for CORM-3. However, another CORM, CORM-A1, which does not contain any transition metal, but boron instead, slowly releases CO. In this work, a new compound was also tested, CORM-371. This CORM contains manganese and liberates CO slowly. While all three CORMs inhibit growth and metabolism of *P. aeruginosa*, as determined by a decreased oxygen consumption, CORM-A1 reduced O_2_ consumption less than did CORM-2 and CORM-3 and only slowed the initiation of bacterial growth. Moreover, CORM-A1 had no bactericidal properties but acted more as a bacteriostatic agent (i.e., it only stopped bacteria from reproducing and it did not kill any bacteria). Although both CORM-A1 and CORM-371 are both slow CO releasers, CORM-371 was found to be intermediate between CORM-2/CORM-3 and CORM-A1 in this respect. It elicited a stronger antibacterial effect and inhibition of O_2_ consumption than did CORM-A1, but the ruthenium-based compounds still showed the largest sustained effect. Owing to the similar rates of CO release from CORM-371 and CORM-A1, but the ability of CORM-A1 to only exert a bacteriostatic effect, the kinetics of CO release from CORMs is not necessary for their antibacterial effects. Instead, as indicated by the results obtained with the ruthenium-based CORM-2 and CORM-3, which rapidly decrease bacterial growth, the presence of a metal center that favours CO targeting bacteria and the nature of transition metal are more likely to be the causes of the antibacterial activity exhibited by these CORMs. A recent investigation by Flanagan et al. has also shown that [Et_4_N][MnBr_2_(CO)_4_] is active against a variety of *P. aeruginosa* strains [24].

Although the best understood targets of CORMs are hemes, Wilson et al. [90] have reported that a CORM can have enhanced toxicity against nonclassical targets, that is, nonheme bacteria. When exposed to *E. coli* hemA mutants and *Lactococcus lactis*, two bacteria that lack heme, the CORMs reduced their growth considerably. CORMs were also found to disturb the cell membranes of these two bacteria and deplete iron levels in wild type and heme-deficient *E. coli*.

## 3. Effect of CORMs in Neuroprotection and Neuronal Differentiation

An investigation by Verma et al. [91] has indicated that CO gas may function as a neurotransmitter. These researchers found high concentrations of heme oxygenase in the brain. Moreover, since heme oxygenase degrades heme to biliverdin and releases CO in the process, there was a substantial amount of CO in the brain as well. It was discovered that CO is a weak activator of guanylyl cyclase (GC) thus allowing CO to effect smooth muscle relaxation and potentially block platelet aggregation. What therefore is the role of CO in neural activity? Two independent research groups have found that CO is linked to the functions of nonadrenergic, noncholinergic neurons of the enteric nervous system and, therefore, has an impact on spontaneous slow waves of the smooth muscle [92, 93]. Furthermore, it has been reported that CO may influence long-term potentiation (LTP) in a tonic manner [94]; through the strengthening of synapses, LTP has been shown to be involved in learning and memory. In addition, endogenous CO has been implicated in attenuating endotoxin-stimulated arginine vasopressin release. Excessive vasopressin release leads to greatly increased water retention by the kidneys and contributes to the dilation of blood; this dilation consequently leads to a low salt concentration in the blood, a condition known as hyponatremia [95]. However, CO may also be associated with pathological states, and a few studies have concluded that CO may be linked to onset of Parkinson's disease [96–98].

Two research teams have examined the effect of exogenous CO on the brain via CO inhalation. In the study by Piantadosi et al. [99], awake rats were exposed to high concentrations (2500 ppm) of CO gas for 60 min. This exposure led to delayed histological damage in the brain, the cerebral cortex being the most affected followed by the basal ganglia and cerebellum. However, the performance of the CO-exposed rats improved gradually and approached that of control rats one month after the exposure. Exposure to high concentrations of CO led to interstitial glutamate accumulation which was attributed to increased hydroxyl radical formation. In addition, CO interfered with mitochondrial redox state and energy production. Glial damage was also seen upon CORM administration. These observed effects led to the conclusion that CO exhibited toxic effects in the brain [99]. Another study confirmed the same finding by investigating the effects of CO poisoning on the brain in the acute and chronic periods using magnetic resonance imaging. After examining the brains of 16 previously CO-exposed patients, the imaging showed necrosis of the globus pallidus, white matter lesions with demyelination or necrosis, lesions of the cerebral cortex, and necrotic lesions of the hippocampus [100]. Again, this study validated the notion of CO poisoning leading to brain dysfunction. However, CO under lower concentrations produced an opposite effect on the brain. The research by Quieroga et al. [101] was aimed at evaluating the effect of CO on astrocyteneuronal communication. The communication between neurons and astrocytes is crucial for neurogenesis, normal dendritic maturation, spine formation, and integration of adult born neurons. Astrocytes play a role in neuroinflammation and neuroprotection—both processes are compromised in diseases, such as Alzheimer's in which there is a substantial amount of neuronal cell death. Upon the addition of 40 *μ*m of t-BHP (a neuronal cell death inducer) on neurons and cocultures (primary cultures of astrocytes and neurons), a considerable amount of neuronal death occurred. Though, once CO was added in the coculture, neuronal survival levels increased. Moreover, CO influenced ATP extracellular content; extracellular ATP is a signaling molecule involved in the communication between astrocytes and neurons. Following CO treatment, intracellular ATP concentration increased. Not only did CO boost ATP content, but it also raised rates of consumption of the amino acids, serine and cysteine, considerably. This result indicated that amino acids were being converted into pyruvate at a much higher rate thus setting in motion the citric acid cycle, which results in oxidative phosphorylation and ATP production. To determine the role of ATP in astrocyte-neuron communication, the ATP extracellular content was measured at different times. The results indicated that CO stimulated ATP metabolism, which in turn triggered increased communication between astrocytes and neurons, therefore causing the astrocytes to protect the neurons from apoptosis.

Because of the increasing occurrence of neurodegenerative diseases and ischemic stroke, balancing the amount of neurogenesis is a hopeful method to improve the detection of the symptoms and ultimately cure those disorders. Almeida et al. [25] have demonstrated that CO promotes neuronal differentiation and increased NT2 neuronal yield by boosting cell metabolism. When the cultures were treated with CORM-A1, the final number of postmitotic neurons was duplicated. Kinetic parameters for cell growth displayed an increase on growth rate, doubling time, and fold increase when CORM-A1 was added into the medium. Moreover, in the presence of CORM-A1, the total number of a mixed cell population (composed of progenitor cells and postmitotic neurons) was higher after 24 days of differentiation than without CORM-A1.

Higher levels of mRNA expression of the specific neuronal differentiation markers nestin, tuj1, and microtubule-associated protein 2 (MAP2) were observed when treated with CORM-A1 by comparison with treatment with retinoic acid alone [25]. This CORM was also shown to modulate cell metabolism by increasing neuronal yield. The expression of mRNA (Glut1 and MCT2), pyruvate dehydrogenase, and lactate dehydrogenase membrane transporters all increased upon administration of CORM-A1. In addition, when supplemented with CORM-A1, the ratio of lactate concentration/glucose concentration was lowered, which validated the conclusion that the differentiation process was elevated owing to a switch from oxidative to glycolytic metabolism that occurred early on.

A promising approach to repair the impaired brain following a hemorrhagic stroke is neural stem cell (NSC) transplantation, but there is reduced cell survival because of iron overload following the transplantation. According to the data of Xie et al. [26], addition of CORM-2 increased the viability of NSC in a concentration-dependent manner, suggesting a neuroprotective effect of the CORM against iron overload. CORM-2 also reduced the number of apoptotic cells, whereas the ferrous iron-treated and iCORM-2 (inactive CORM) groups increased it. Iron overload generated nuclear translocation of NF-*κ*B p65 in neural stem cells but when CORM-2 was administered, the nuclear translocation was diminished. Additionally, the levels of the molecules, which induced apoptosis (Bax and cleaved caspase-3), were elevated when exposed to excess iron. Treatment with CORM-2 attenuated the levels of these molecules to half that of the FeCl_2_ group. As well as investigating the viability of transplanted neural stem cells, Wang et al. [27] examined the effect of CO treatment on brain mitochondrial impairment and brain injury in rats. Following the return of spontaneous circulation after cardiac arrest or return of spontaneous circulation (ROSC), the introduction of CO resulted in an increase in the 3-day survival time of the rats. After 24 hours in ROSC-challenged rats, CORM-2 treatment led to an elevated neurologic deficit score when measuring arousal, reflex, motor, sensory, and balance responses. Furthermore, CO was observed to lower S-100B levels; S-100B refers to a marker of neurological outcomes after cardiac arrest. CO supplementation also elevated ATP levels, the mitochondrial respiratory ratio, ΔΨ*m*, and mitochondrial DNA; these factors are all indicators of mitochondrial function, suggesting that CO lessens mitochondrial damage associated with ROSC [27].

A different type of CORM, known as CORM ALF492, has been shown to fully protect mice against experimental cerebral malaria and acute lung injury via controlling CO delivery *in vivo* without affecting O_2_ transport by hemoglobin. Through altering the ligand in the Ru-core scaffold and metal-ligand linkage, Pena et al. [29] have managed to synthesize CORM ALF492. This CORM has a galactose-derived ligand coordinated to the Ru center via a thioether linkage, which helped to improve water solubility and compatibility with biological molecules. The presence of the galactose ligand may also allow an elevated degree of specificity by targeting appropriate receptors in the liver. Owing to its increased solubility in water and high level of specificity, CORM ALF492 was able to protect mice from death due to cerebral malaria, which is mainly caused by the parasite, *Plasmodium falciparum* [29]. CORM ALF186, was also able to prevent nerve cell apoptosis in a simulation of ischemic insult to neuronal cells. When SH-SY5Y-cells were exposed to rotenone to simulate ischemic respiratory arrest, only CORM ALF186 and not the CO-free inactivated ALF186 (iALF 186) prevented nerve cell apoptosis induced by rotenone by increasing cellular cGMP levels and sGC expression [28]. Furthermore, based on the study by Woo et al. [30], CO was found to increase the expression of oxidative stress-protecting enzymes within the developing inner ear (cochlea) in a mouse model and thus demonstrated that mild CO exposure may be beneficial for diseases caused by oxidative stress.

Excessive inflammatory responses can be harmful and the modulation of inflammation in microglia by CO has been shown to be necessary for the control of neuroinflammation. Several studies concerning the anti-inflammatory effect of CO have been performed in vitro using BV-2 microglial cells. Bani-Hani, for example, has found that CORM-3 decreases NO production and TNF-*α* release in response to LPS, thrombin, and IFN-γ stimuli, thereby limiting the inflammatory response of neurons [31]. Lin et al. have also recently reported that CORM-3 sequentially activates a c-Src/Pyk2/PKC*α*/Erk1/2 pathway in rat brain astrocytes causing induction of HO-1 and subsequent suppression of interleukin-1*β*-mediated neuroinflammation [32].

## 4. Contributions of CORMs in Nociception and Diabetes

Painful neuropathy is the most common complication of diabetes mellitus affecting 40% of people with type-1 diabetes. Administration of cannabinoid receptor 1 (CB1R) and 2 (CB2R) agonists does reduce pain in many animal pain models and may be a target for treatment of neuropathy. In a mice model of streptozotocin- (STZ-) induced diabetic neuropathy, a study was undertaken to evaluate the antinociceptive effects of the CB2R agonist, JWH-015, when combined with CORM-2 and when with CORM-2 alone. A STZ injection induced mechanical allodynia (pain due to stimuli that do not normally provoke a painful response), thermal hyperalgesia (increased sensitivity to pain), and thermal allodynia (pain from normally mild skin temperatures in the affected area). Upon administration of CORM-2, mechanical allodynia, thermal hyperalgesia, and thermal allodynia were significantly attenuated compared to the control group. Furthermore, low levels of HO-1, CB2R, and overexpression of nitric oxide synthase (NOS1) are characteristic of diabetes. Supplementation of CORM-2, combined with JWH-015, increased protein levels of HO-1 and CB2R and decreased the NOS1 protein levels. Taken together, these results suggest that CORM-2 could act as a potential treatment for painful neuropathy [33].

Additional evidence of CORMs being able to treat neuropathic pain has come from a study in which the antinociceptive effect of CORMs was evaluated in a murine model of sciatic nerve injury [34]. Sciatic nerve injury elevates NOS1, NOS2, and CD11b/c protein levels and increases spinal microglial activation; however, expression of these proteins and activation of microglial cells were attenuated upon CORM-2 administration. It was also observed that CORM treatment reduced the mechanical and thermal hypersensitivity caused by sciatic nerve injury. Next, the effects of CORM-A1 on preventing diabetes via beta cell regeneration were examined by Nikolic et al. [35]. In diabetes, the immune system of the body attacks and destroys the beta cells; this situation is characterized by a high interleukin and T-helper cell count as well as a low TGF-ß count and Ki-67 protein expression. When mice were treated with CORM-A1, the frequency of T-helper cells was noted to be considerably lowered in the spleen and pancreatic-draining lymph nodes of the mice when compared to the values for the non-CORM-A1-treated control group. CORM-A1 also resulted in an elevation in TGF-ß production and Ki-67 protein expression; hence, it counteracts the effects of diabetes.

Another study was undertaken to investigate the ability of CO in preventing diabetes via injecting dendritic cells treated with CO into diabetic rats [102]. This approach resulted in an inhibition of diabetes independent of IL-10 secretion and CD4+ T cell expression in dendritic cells; thus, this denoted that the protection was not mediated by dendritic cells (DCs), but instead was mediated by CO.

## 5. Role of CORMs in Inflammatory Disease

The CO gas liberated by CORMs also has an important role in modulating inflammation, a symptom observed in multiple pathological conditions. An example of such a pathological condition is colitis, which is described as an inflammatory reaction in the colon. One study exposed NIH 3T3 fibroblasts, cells that contribute to colitis, to varying concentrations of CORM-2 for 24 hours [36]. A significant decrease in cell survival was noted suggesting an anti-inflammatory effect of CORM-2 on colitis-inducing cells.

Another study has focused on the potential uses of a new type of nanosized CO donor, CO-HbV (CO bound hemoglobin encapsulated liposomes), with respect to providing therapeutic benefits for the symptoms of colitis [37]. CO-HbV administration was seen to suppress the progression of symptoms associated with colitis as well as prolonging the survival duration of dextran sulphate sodium- (DSS-) induced mice. Tissue damage, such as necrosis and ulcers, was found in DSS-induced colitis mice, whereas DSS mice that were treated with CO-HbV displayed lower amounts of tissue damage and a histological appearance that was like that of the control mice. Similarly, the administration of DSS generated anemia due to bleeding in the colon; reduced levels of red blood cells (RBC) and hemoglobin (Hb) were observed in the DSS-induced colitis mice. In contrast, the CO-HbV treatment overturned this reduction in RBC and Hb. Therefore, the overall results from the two studies described here [36, 37] support the notion of CORMs being able to negate cell death and the harmful effects induced by colitis. Certain inflammatory diseases work by targeting dendritic cells thus resulting in an inflammatory response. The roles of dendritic cells are to recognize, destroy, and present harmful antigens to T-cells; hence, they protect the host from various infections. When exposed to lipopolysaccharide (LPS) derived from bacteria, however, the dendritic cells undergo an inflammatory response via binding to toll-like receptor 4 (TLR4)/myeloid differentiation factor-2 (MD2) complex present on the surface of the cell.

Research undertaken by Riquelme et al. [38] showed that CO was able to prevent LPS-mediated inflammation. The expression of TLR4/MD2 on the surface of dendritic cells was reduced upon CO treatment, whereas in the control cells, no changes were observed. Moreover, after CO exposure, mice that received LPS preserved their ability to move with no changes in their velocity. Conversely, the groups of mice that received LPS and were subjected to iCORM-2 displayed a decrease in their mobility, with values falling more than 70% compared with the values noted before LPS exposure. LPS mice supplemented with either iCORM-2 or CORM-2 maintained their body weight and had an increased survival rate. However, mice treated with iCORM-2 and then challenged with LPS experienced a decrease in body weight by almost 18% and had low survival rates. Moreover, CO treatment was found to protect mice from the toxic increase of neutrophils in blood associated with septic inflammation. Mice treated with iCORM-2 and then exposed to LPS demonstrated a 25-fold significant increase in neutrophil count. This increase in neutrophils was not seen in LPS mice exposed with CORM-2.

On a molecular level, how do CORMs mediate inflammation? Research conducted by Jung et al. [103] investigated these mechanisms, and the contribution of CO to NLRP3 (nucleotide-binding domain, leucine-rich-containing family, pyrin domain-containing-3) inflammasome activation in macrophages was explored. Inflammasomes are protein complexes in the cytosol that cause cleavage of caspase-1, which results in the maturation and secretion of proinflammatory cytokines, such as interleukin-1 (IL-1) and IL-18. This cascade of events consequently leads to inflammation in the body. The study found that CO treatment inhibited the secretion of IL-1 and IL-18 in response to LPS and ATP in macrophages by preserving the mitochondrial membrane potential. Moreover, mitochondrial ROS generation induced by LPS and ATP was inhibited by CO treatment. CO also inhibited mtDNA translocation into the cytosol, which is associated with the obstruction of cytokine secretion. Therefore, these findings demonstrate the ability of CO to reduce inflammation via negatively regulating LRP3 inflammasome activation in macrophages.

Yeh et al. [39] have also evaluated the molecular mechanisms of anti-inflammatory effects of CORMs. It was hypothesized that CORMs could promote NF-*κ*B-p65 glutathionylation. When the study was conducted, however, CORMs were found to repress TNF-*α*-induced monocyte adhesion to endothelial cells and reduce the expression of ICAM-1. Furthermore, CORMs were noted to block NF-*κ*B-p65 nuclear translocation. CORMs also induced Nrf2 activation and HO-1 expression, which elevated p65 glutathionylation. Therefore, these findings indicate that the glutathionylation of p65 is responsible for the NF-*κ*B inactivation mediated by CORMs.

A common phenomenon associated with inflammatory diseases is excess blood clotting. The effect of CORM-2 on blood coagulation was investigated by testing it on the expression of TF and PAI-1 and on signaling pathways (MAPK and NF-*κ*B), which all stimulate thrombosis [40]. It was demonstrated that CORM-2 subdued TNF-*α*-induced TF and PAI-1 upregulation in HUVECs (human umbilical vein endothelial cells). Moreover, CORM-2 treatment suppressed MAPK and NF-*κ*B signaling pathways activation by TNF-*α*. Based on these results, CORM-2 supplementation was seen to decrease blood clotting induced by inflammation.

According to Fagone et al. [41], CORMs may also pose an effective treatment for yet another inflammatory disorder—uveitis, the leading cause of blindness due to an inflammation of the eye. CORM-A1 was noted to improve the morphology of the retina in the eye. Furthermore, rats treated with CORM-A1 expressed lower levels of IFN-gamma and IL-17A and increased amounts of IL-10 when compared to the non-CORM-treated uveitis-induced mice.

An additional complication due to inflammatory diseases is the onset of chronic inflammatory pain. Negrete et al. [42] found that CORM administration reduced this pain. When exposed to CORM-2, the inflammatory pain symptoms induced by complete Freund's adjuvant in mice, which consist of mechanical allodynia and thermal hyperalgesia, were considerably reduced. Moreover, inflammatory pain induced enlarged NOS1 protein expression in mice. This upregulation was prohibited by CORM-2 treatment in only wild-type cells; it did not decrease NOS1 levels in the NOS2-knockout mice.

Intestinal epithelial cells (IECs) play a key role in protecting against pathogens in the intestinal tract as well as maintaining gut barrier function. However, when inflamed, the function of the cells is greatly reduced, making them more vulnerable to microbes. Mu et al. have observed that CORM-2 treatment results in an improvement of the barrier function of IEC-6 cells [43]. In addition, the CO released from CORM-2 inhibited the secretion of proinflammatory cytokines (TNF-a and IL-1b) produced by LPS. One of the underlying mechanisms of inflammation includes the induction of myosin light-chain phosphorylation in the IECs. CORM-2 suppressed this phosphorylation in the cells; taken together, these data suggest that CORM-2 carries the ability to restore function of the IECs in the instance of inflammation.

Periodontal disease, yet another example of an inflammatory disease, has been linked to other potential disorders, such as cardiovascular disease, diabetes, and stroke, and a study by Choi et al. [44] has concluded that CORM-3 possesses anti-inflammatory activity against the periodontal-disease inducing the pathogen *Prevotella intermedia.* Upon the addition of CORM-3, HO-1 expression in LPS-exposed cells was enhanced. Although LPS-induced phosphorylation of p38 and JNK and the degradation of I*κ*B-*α* were not affected by CORM-3, nuclear translocation of NF-*κ*B p65 and p50 subunits was blocked by CORM-3 in LPS-treated cells. This result is significant due to the activation of NF-*κ*B being associated in inflammation and disease. In addition, CORM-3 was noted to diminish LPS-induced p65 and p50 binding to DNA and phosphorylation of STAT1.

What may be the main underlying cause of several inflammatory disorders? According to Patterson et al. [45], polymorphonuclear leukocyte-derived myeloperoxidase (MPO) is known for its direct peroxidation as well as generation of potent oxidizing compounds, namely hypochlorous acid, which contributes to increased oxidant damage, which in turn leads to tissue injury and inflammation. These researchers found that CORM-3 suppresses MPO oxidative activity. The inactivated CORM-3 (iCORM-3) was also tested, and it showed no attenuation in the MPO activity, suggesting that binding the released CO to the MPO heme is crucial for its effects. When the activity of MPO in peroxidation and halogenation was tested, CORM-3 showed an inhibitory effect in both cases. Surprisingly, iCORM-3 also was able to decrease the chlorination activity of MPO, but to a lesser extent when compared to active CORM-3. These may be due to a CO-independent downstream reactivity of the iCORM metal centers with hypochlorite.

## 6. Role of CORMs in Sepsis: A Systemic Inflammation

As mentioned by Woo et al. [30], oxidative stress is believed to contribute to tissue damage, and nitrosative stress is often suggested to be initiated by an inflammatory cascade that consists of acute phase protein synthesis, upregulation of inflammatory adhesion molecules, and proinflammatory cytokine liberation. Severe injury is concerned with lipid peroxidation mediated by reactive oxygen species (ROS) and NO; lipid peroxidation is known to be an essential cause of oxidative damage to cellular membranes. In another study, Sun et al. [46] found that upon *in vivo* administration of CORM-2, the protein expression of inducible NOS and the overabundance of NO were considerably attenuated in thermal injury-induced septic mice. Furthermore, both the production of ROS and NO were decreased in LPS-stimulated HUVECs when incubated with CORM-2. In addition, recent findings suggest that the stress-inducible gene HO-1 is an important factor in protecting against oxidative stress. Sun et al. have also shown that the induction of HO-1 effectively provides protection both *in vivo* and *in vitro* against oxidative stress. They found that HO-1 is upregulated in HUVECs by LPS stimulation, and they went further by testing it with CORM-2. Their results indicated that not only was LPS able to induce the expression of HO-1, but also that an increase in HO-1 expression can be enhanced by CORM-2 treatment. Hence, the study concluded that CORM-2 ultimately leads to cytoprotection and inhibition of oxidative stress during sepsis.

When mice were impaired with cecal ligation and puncture (CLP), which led to systemic inflammation known as sepsis, CO was found to increase survival via strengthening the processes of autophagy (degradation of harmful or damaged intracellular contents) and phagocytosis (engulfment of bacteria by cells) [48]. Treatment with CO induced the expression of the autophagy proteins, beclin 1, Atg7, and LC3B; CO was also observed to increase the number of autophagosomes, the structure in which autophagy occurs, in the lungs of mice. Further, CLP-induced Becn1+/+ mice (mice with the eclin protein) that were then treated with CO demonstrated increased survival compared with the room air-exposed control mice. Next, the ability of CO to mediate phagocytosis was assessed. It was noted that CO elevated the phagocytic activity of bone marrow-derived macrophages induced with LPS. The process by which CO can increase phagocytosis was revealed by its observed ability to induce SLAM (a signaling molecule responsible for killing bacteria by macrophages) mRNA expression in bone marrow derived-macrophages.

An additional study that focused on the role of CO in sepsis was undertaken by Zhang et al. [47], who discovered that CORM-2 could protect intestinal epithelial tight junctions when damaged with LPS in a rat CLP model. CORM-2 improved the morphology of the intestinal mucosa during sepsis when compared to CLP alone or treatment with iCORM, which showed ulceration and hemorrhage on the mucosa. Additionally, CORM-2 was seen to improve CLP-induced, tight-junction disruption and increase the proximity of the intercellular connection. Not only did CORM-2 ameliorate the physical appearance of the tight junctions, but it also raised the expressions of occludin, claudin-1, and ZO-1 proteins; in contrast, these proteins were observed to be reduced during sepsis. By improving the morphology of tight junctions and increasing tight junction protein levels, the study established that CORM-2 could protect the LPS-induced damaged intestinal tight junctions.

A common complication induced by sepsis is known as AKI (acute kidney injury), and, based on the results of Nagao et al. [37], CO was found to reverse the harmful effects of AKI. Blood urea nitrogen and serum creatinine, frequently used markers for AKI onset, were increased following CLP-induced sepsis. In contrast, when treated with CORM-2, levels of these markers were decreased. Sepsis with AKI resulted in a greater mortality, killing about 50% of the rats within three days. However, upon CORM-2 administration, the rats were seen to possess a higher longevity. Through its capability of decreasing markers for AKI onset and increasing survival rates, CORM-2 can be noted to counteract AKI-induced toxicity.

In yet another examination of the role of CO in sepsis, Liu et al. [51] have noted that CORM-2 reversed the LPS-induced activation of platelets. The LPS groups had abnormal platelet function, namely, spreading and aggregation. In the CORM-2 group, however, the platelets were only mildly activated; CORM-2 treatment reduced spreading as well as aggregation. To resolve whether LPS induced platelet secretion, LPS-induced ATP release was inspected in human platelets. LPS stimulation increased ATP release; in contrast, CORM-2 treatment decreased ATP release in response to LPS stimulation. Furthermore, while the platelet membrane glycoproteins, GPIb*α* and GPVI, were upregulated in sepsis, CORM-2 decreased glycoprotein upregulation. CORM-2 treatment also inhibited LPS-induced HS1 phosphorylation, which is also linked to platelet activation. Furthermore, the effect of CORM on hepatic mitochondria and abnormal glucose metabolism in septic mice was evaluated [52]. With respect to the histopathological changes of the liver, CLP-induced mice suffered hepatocellular damage and swelling; in contrast, CLP mice that later received CORM-2 treatment demonstrated less severe liver damage. Hyperglycemia, a condition associated with sepsis, is concerned with high activities of glucose metabolism. The CLP group of mice showed an increase in the level of hepatic glucose metabolism and GK, an enzyme involved in the metabolism; these functions were suppressed by CORM-2 treatment. Plasma alanine transaminase (ALT) and aspartate transaminase (AST) levels were utilized to indicate the degree of hepatocellular damage. Although the CLP group of mice exhibited higher activities of ALT and AST, the CLP + CORM-2 group reduced those levels. Another indicator of sepsis, lactic acid, was considered: the CLP and CLP + iCORM groups had increased levels of lactic acid, whereas the CLP + CORM group had lower levels of the acid. The results from the study also indicated that CORM-2-exposed mice had higher survival rates than the septic CLP-induced mice.

CORM-3 has also recently been reported to alleviate myocardial dysfunction due to sepsis as well as to provide therapeutic benefit for sepsis in lung injury [50]. In the case of the former, CO released by CORM-3 is proposed to inhibit the activation of the NLRP3 inflammasome in cardiac fibroblasts preventing myocardial apoptosis. In the latter, CORM-3 restored annexin A2 (a membrane-associated protein involved in fibrinolysis homeostasis which is downregulated in LPS-induced sepsis) to normal levels when introduced intraperitoneally. While this observation was ascribed to the presence of CO, it is not entirely conclusive since the influence of the inactivated CORM was not investigated [49].

A substantial recruitment of polymorphonuclear (PMN) leukocytes to the affected organs is another important characteristic associated with severe sepsis [53]. Exposure of the hCMEC/D3 cell line with LPS resulted in an increase in PMN rolling and adhesion upon fluid shear stress. Treatment of hCMEC/D3 with CORM-3 was seen to suppress both LPS-induced PMN rolling and adhesion to hCMEC/D3. Stimulation of hCMEC/D3 with LPS resulted in upregulation of cell adhesion molecules, including eselectin, ICAM-1, and VCAM-1 expression. Upon the addition of hCMEC/D3 with CORM-3 but not with iCORM-3, VCAM-1 expression was effectively reduced. However, CORM-3 expression failed to reduce LPS-induced expression of E-selectin and ICAM-1. Although CORM-3 was able to decrease leukocyte infiltration and attenuate expression of some of the proteins expressed in sepsis, it was not able to modulate them all; this indicates that whether CORM-3 may be an effective treatment for sepsis is questionable based on these results.

## 7. Obesity Affected by CORMs

Chronic obesity, identified with increased inflammation, increases the risk of health problems such as diabetes, heart disease, and arthriti, and the main objective of the study by Hosick et al.co [54] was to investigate whether chronic treatment with CORM-A1 could reverse dietary-induced obesity, hyperglycemia, and insulin resistance. The study used mice that were divided into different treatment groups: (i) a control high-fat diet group; (ii) a group receiving a fat diet and saline intraperitoneal injection; (iii) a group exposed to a fat diet and CORM-A1; and (iv) a last group with a fat diet exposed to iCORM. CORM-A1 treatment caused a lack of weight gain in the high-fat-induced mice over the first 18 weeks of the study compared to other groups; over the last 12 weeks of the study, the mice started to lose weight, up to 33% of their initial body weight. Not only did CORM-A1 induce fat reduction and weight loss, but it also provided a 45% increase in lean body mass as a percent of total body weight at 30 weeks. In addition, fasting blood glucose and plasma insulin levels were elevated in all groups before treatment. However, CORM-A1 treatment resulted in a considerable reduction of hyperglycemia after 6 weeks of treatment, which continued until the end of the study. After 30 weeks of treatment, the fasting blood glucose levels in the CORM-A1-treated mice were 55% of those observed in the control mice. Moreover, CORM-A1 treatment resulted in a significant decrease in plasma insulin levels as compared to all other groups after both 24 and 30 weeks of treatment. HMGB1 is a protein responsible for high levels of inflammation that is linked to obesity. Following CORM-A1 treatment, HMGB1 levels were reduced in dietary-induced obese mice in comparison to those found for the other groups suggesting an antiinflammatory effect induced by CORM-A1 on obesity.

Zheng et al. [55] have also studied the effect of CORM's on obesity. They discovered that obesity may be due to leptin resistance caused by endoplasmic reticulum (ER) stress. Leptin is a hormone that circulates in the bloodstream and serves the function of regulating food intake and body weight. They hypothesized that CO could prevent leptin resistance during ER stress. Thapsigargin or tunicamycin was used to cause ER stress in human cells, and they were found to hinder leptin-induced STAT3 phosphorylation, establishing that ER stress induces leptin resistance. CORM-2 treatment of these cells blocked the STAT3 phosphorylation and induced the phosphorylation of protein kinase R-like ER kinase and eukaryotic translation initiation factor-2 during ER stress. When tested *in vivo*, CO exposure reduced the body weight of animals being fed diets high in fat. Based on the results of the two studies, it is evident that CO does have a role in attenuating obesity.

## 8. Effect of CORMs on Angiogenesis, Aggregation, and Cancer

According to Ahmad et al. [56], CORM-2 halts angiogenesis caused by the vascular endothelial grown factor (VEGF). When exposed to VEGF, endothelial cells experience an increase in actin stress fiber production indicated by specific spindle-like processes. This production was significantly inhibited by CORM-2, but not as much by iCORM-2. Moreover, CORM-2 prevented VEGF-dependent endothelial cell migration and proliferation. The retinoblastoma protein (Rb) is a tumour suppressor protein whose purpose is to restrict the ability of the cell to progress through the cell cycle. In this way, Rb prevents over replication in cells and hence cancer. However, when Rb is continuously phosphorylated on serine residues, as is the case in VEGF, it can no longer suppress the cell cycle and, as a result, the cell replicates in excess. CORM-2 was noted to attenuate VEGF-induced phosphorylation of Rb and, in this way, it was able to stop extreme cell replication and therefore angiogenesis caused by VEGF. Another study by Fayad-Kobeissi et al. [57] focused on examining the biochemical and potential anticancer properties of CORM-401, a novel CO-releasing molecule containing manganese in its metal center. CORM-401 was observed to promote vasorelaxation of precontracted aortic rings (3 times higher than that evoked by CORM-A1), and this was enhanced in combination with H_2_O_2_. In addition, CORM-401 was found to exhibit proangiogenic properties, as determined by augmented gene expression of VEGF and IL-8. Furthermore, as demonstrated by Loureiro et al. [58], folic acid-tagged protein nanoemulsions prefer to be internalized on the B-cell lymphoma cell line (A20 cell line). The folic acid-tagged protein nanoemulsions that contained CORM-2 resulted in an increased antitumor activity and increased the survival of mice with A20 lymphoma tumours.

Pancreatic fibrosis is associated with pancreatitis and pancreatic cancer; pancreatic stellate cells (PSCs) are mainly responsible for the development of fibrosis [59] and, owing to its ability to induce cell cycle arrest, CORM-2 can prevent global protein synthesis in PSCs. PSCs were found to induce a decrease in the amounts of both phosphorylated eEF2 and eIF2 (components of the translational machinery) as well as an increase in the quantities of phosphorylated eIF4E and 4E-BP1. Exposure of CORM-2 to these cells resulted in no effect on the phosphorylation of eIF2 or eIF4E, but rather it prevented the decrease of phosphorylated eEF2 caused by the cells and reduced the level of phosphorylated 4EBP1. Next, CORM-2 was tested as to whether it affects translation in PSCs. Effectively, CORM-2 treatment resulted in a 51% reduction in protein synthesis. This repression led to further testing of CORM-2 on its effect with regards to the proliferation of PSCs; the levels of cyclin and phosphorylated Rb, both of which cause proliferation, were compared. The results indicated that CORM-2 was able to abrogate the increase in cyclin D1 and cyclin E and Rb phosphorylation induced by PSCs. An important property of cancer cells is their ability to aggregate. Integrins are cell adhesion receptors capable of modulating rapid adhesion and deadhesion events, without altering the number of molecules expressed. Ligand interactions with integrins suggest integrin-dependent cell adhesion, which is regulated by multiple signaling pathways initiated by other cellular receptors. This signaling allows for fast leukocyte arrest on endothelium, cell migration, and mobilization. *α*4*β*1-integrin (CD49d/CD29, Very Late Antigen-4, VLA-4) is expressed on leukocytes, dendritic cells as well as cancer cells. When a CORM was tested on VLA-4, the results demonstrated that the CORM induced a rapid decrease in the binding of the VLA-4-specific ligand [61]. Moreover, exposure to the CORM prevented VLA-4/VCAM-1-dependent aggregation. These findings suggest that CORMs seem to possess the capability to avert aggregation, a striking feature of cancer cells.

Photochemotherapy is a promising strategy for the treatment of several types of cancers. PhotoCORMs, which can release CO once activated by light, as mentioned by Tinajero-Trejo et al. [18], can be efficiently internalized in HT29 human colon cancer cells, as demonstrated by Niesel et al. [60]. Based on the study, no change in the number of cells was observed with the control group nor with the cells incubated with PhotoCORM in the dark for up to 48 hours. However, when irradiated at 365 nm for 10 min in the middle of the incubation period, PhotoCORM treatment led to a considerable reduction in cell biomass, whereas direct cell damage by irradiation in the control group displayed a relatively smaller reduction.

## 9. Contribution of CORMs in Hemorrhagic Shock and Postresuscitation Injuries due to Transplantation and Surgical Operation

Hemorrhagic shock is a condition that results when an insufficient amount of oxygen and nutrients is delivered to the tissues and cells due to certain injuries such as to the liver and kidney. Resuscitation, the process of correcting injuries, can potentially result in even more trauma to the patient (e.g., CPR can induce broken ribs) [104]. Nassour et al. [62] have developed a surgical rat model for hemorrhagic shock which is induced by withdrawing blood and returning it back to the animal to maintain a relatively low blood pressure. After the shock, mice were resuscitated with Ringer's lactate solution using two times the volume of maximum shed blood. The control group of mice had the operation performed but did not undergo the resuscitation. The noncontrol group had CORM-421 intravenously administered with the Ringer solution during the resuscitation. Hemorrhagic shock and resuscitation (HS/R) contributed to a reduction in normal endothelium fenestrations, rounding of cells, and increased adherent leukocyte count in mice not exposed to the CORM. When the CORM was delivered to the rat, it was observed that the number of fenestrations, cells, and adherent leukocytes was maintained owing to the ability of the CORM to reduce the expression of HS/R-induced cytokines. Hence, CO managed to protect against HS/R in a rat model. In addition, when CORM-3 was administered to HUVECs, which were subjected to 20 hours of cold storage, an increased frequency of live cells, reduced apoptosis, and decreased mitochondrial transmembrane potential in HUVECs was noted [63]. This result was in direct contrast to the control group, which displayed an opposite effect. Regarding the morphology of the renal tissue, a reduction in glomerular atrophy and necrosis was observed in the CORM-3 group versus the control group. Furthermore, on a molecular level, CORM-3 exposure led to a 2.1-fold upregulation in Bcl-2 gene expression, a gene involved with regulating apoptosis. One interpretation of this study is that CO increases the viability of cells in cold storage for future transplantation.

In another study, CORMs were examined by Yao et al. [64] in a rat model to determine whether they can offer protection of the heart from postresuscitation myocardial injury and cardiac mitochondrial dysfunction. The rats were induced with ventricular fibrillation and then resuscitated; it was found that one hour following resuscitation, the mean aortic pressure, ventricular contractility assessment, and isovolumetric contractility values were increased in the group exposed to low concentrations of CORM-2 compared to the control group. In terms of the myocardium morphology, 3 hours after resuscitation, the control group displayed myocytolysis and transverse contraction bands. Rats exposed to CORM-2 depicted less severe myocytolysis and relief from damaged myocardial fibers. Additionally, the CORM-2-exposed rats showed decreased production of cardiac mitochondrial ROS indicating that CORM-2 reduced oxidative stress in the resuscitated rats by uncoupling mitochondrial respiration. However, high concentrations of CORM-2 caused the reverse effect; here, there is increased mitochondrial ROS generation, most likely because of its excessive uncoupling action. Taken together, these findings imply that CORM-2 lowers oxidative stress in the heart and ameliorates cardiac function after hemorrhagic shock and resuscitation.

The bioactive properties of CORM-3 have been investigated using two models: an in vitro model of hypoxia-reoxygenation and oxidative stress and a cardiac transplant rejection model [65]. In the first model, it was found that CORM-3 conserved cell viability against reoxygenation-induced damage in a concentration-dependent manner. This cytoprotective effect was observed when CORM-3 was applied to the cells either during the hypoxic event or at reoxygenation. The inactive compound (iCORM-3) did not demonstrate any protection against hypoxia-reoxygenation; this suggests that CO released from the ruthenium carbonyl is necessary to exert the observed effect. For the second model, CORM-3 was seen to prevent cardiac allograft rejection. This was evidenced by the prolonged survival time of hearts transplanted into rats exposed to CORM-3; all hearts were still beating 18 days after the transplantation, whereas the non-CORM-treated mice were subjected to rejection within 9 days of transplantation.

Similar findings by Clark [65] and Musameh et al. [66] have demonstrated that CORM-3 protected isolated hearts against the combined effects of cold ischemic storage/preservation and warm reperfusion injury when transplanted into mice. A higher *dP*/*dt* recovery, which represents increased diastolic function, was seen in hearts treated with CORM-3 when compared with iCORM-3, suggesting that CO release is necessary for enhanced diastolic function of the heart. After a cold storage lasting both 4 and 6 hours, the coronary flow rates were similar at both times with CORM-3-treated hearts, whereas there was a decrease in coronary flow when the hearts were exposed to iCORM-3 after 6 hours when compared to the flow after 4 hours. Owing to the CORM-3-induced persistent blood flow supplying the hearts, they survived for prolonged times, thus demonstrating a successful transplantation in mice.

Caumartin et al. [67] have investigated the ability of pretreating the kidney donor with CORM-2 to prevent ischemia—reperfusion injury in a transplant rat model. The histological analysis of the kidney grafts demonstrated that CORM-2-treated kidneys had decreased damage when compared to the control group (no CORM-2 treatment) postoperation. Ten days after the transplantation, infiltrated lymphocytes and tubular necrosis were observed in the control group, whereas the CORM-2-treated rats exhibited entirely normal histology. On day 70, CORM-2-administered animals showed optimal renal function but an excess accumulation of lymphocytes (indicating an infection or inflammation) and atrophy of the glomerulus. This observation reveals that although CORM-2 administration was noted to lead to a successful kidney transplantation for the first several days, it can have negative effects over a much longer term. Because of this result, the water-soluble CORM-3 was tested as well. The CORM-3-treated mice were only followed for 3 days; at the third day of posttransplantation, improved renal histology and function were noted. Because CORM-3 was not tested for as long a time as CORM-2, unfortunately, no definitive conclusion about the benefit of CORM-3 on posttransplantation survival could be reached.

## 10. Effect of CORMs in Gastric, Intestinal, Kidney, and Liver Disorders

CORMs have been shown to improve lesions and ulcerations found in various parts of the body, and Magierowski et al. [68] have found that the administration of alendronate, despite serving as an inhibitor of osteoclast-mediated bone resorption, can result in gastrointestinal complications including bleeding erosions. The gastric mucosa of rats, pretreated with alendronate, induced damage to the mucosa. In rats preexposed to mild WRS (mild stress by water immersion and restraint in cold water), administration of alendronate produced various gastric hemorrhagic dot-like erosions and band-like lesions. However, CORM-2 treatment diminished mucosal lesions in rats preexposed to mild WRS with subsequent alendronate administration. Moreover, administration of acetic acid to mice was seen to result in the development of deep gastric ulceration in the abdomen. In contrast, mice treated with an aqueous CO gas-saturated solution demonstrated a smaller gastric ulcer, and their wounds were reepithelialized [105].

Excess consumption of ethanol invokes liver injuries in biological organisms. In the study by Bakhautdin et al. [69], the hypothesis that CORM treatment can protect ethanol-induced liver injury was investigated. Exposure to CORM-A1 was observed to reduce hepatocyte cell death, indicated by decreased build-up of CK18 cleavage products and lowered RIP3 expression in hepatocytes. CORM-A1 administration also decreased the effects of ethanol on plasma ALT and AST. Notably, CORM-A1 had positive effects on mRNA regulation for inflammatory mediators, as assessed by its ability to decrease TNFa, IL6, and CXCL10 expression. Moreover, although ethanol exposure increased Ly6C+ cell count, a marker of penetrating immune cells in liver, CORM-A1 reduced this effect. Additionally, CORM-A1 decreased the number of inflammatory foci observed on hematoxylin and eosin sections. The growth of c-ketoaldehydes adducts, such as isolevuglandins (iso[4]LGE2) adducts, in the liver suggests oxidative stress. Ethanol supplementation increased the accumulation of iso[4]LGE2 protein adducts; however, CORM-A1 decreased the build-up. Owing to these results, it was deduced that CORM-A1 could prevent hepatocyte death when the liver is compromised by an increased ethanol intake. However, more recent work by Mangano et al. suggests that CORM-A1 may also possibly be applied for the treatment of autoimmune hepatitis owing to its ability to significantly reduce mortality in a murine model of this disease [70].

Not only does CO reduce liver damage, but it also demonstrates protective effects against nephrotoxicity, an adverse effect caused by the chemotherapy agent cisplatin (CP). When confluent renal tubule epithelial cells (LLC-PK1) were exposed to CP for a period of 16 h, an increase in caspase-3 activity was observed [71]. This effect was followed by cell damage—there was an increased number of cells floating in the culture media. Upon the addition of CORM-3 at concentrations of 1–50 *μ*M, the increase in caspase-3 activity was abolished in a concentration-dependent fashion, suggesting its protective action on CP-induced cells. When CORM-3 was added to cells in the presence of an inhibitor of the GC pathway, the caspase-3 activity was not suppressed. This observation indicated that the protective effect of CO appears to be mediated by cGMP. However, biliverdin, which is also generated along with CO during the degradation of heme by HO, did not have any effect on CP-induced caspase-3 activation, demonstrating that CO specifically is necessary to prevent the nephrotoxicity. When tested *in vivo*, CP caused an increase in plasma urea and creatinine levels. This effect was completely prevented by CORM-3; the plasma urea creatinine levels were reduced to similar ones to those for the control group. Unlike CORM-3, iCORM-3 was unable to improve the renal impairment caused by CP. When analyzed histologically, the CP-treated kidneys displayed severe tissue damage that primarily affected the tubules, whereas the glomeruli structure was maintained. On the other hand, CORM-3 ameliorated the tubular tissue, and its appearance resembled to that of the control kidneys. Furthermore, CP exposure resulted in the apoptosis of kidney cells, whereas addition of CORM-3, but not iCORM-3, prevented the CP-induced apoptosis. Also, CP-treatment caused a 1.2% weight loss in the kidney; this was counteracted by CORM-3, but not by iCORM-3. Furthermore, recent findings have confirmed the *in vitro* renoprotective effect of CORM-3 in normal and cancerous human renal cell lines when subjected to CP-induced toxicity and ischemia-reperfusion injury [72].

Another severe condition is postoperative ileus, known as a temporary impairment of bowel motility that can commonly occur following a major abdominal surgery. The severity of postoperative ileus was noted to be reduced in mice exposed to CORMs; this was evidenced by a partial restoration of intestinal contractility and, hence, a less distended bowel [73]. Three groups of mice were examined: one group had an intestinal manipulation (IM) procedure performed to induce the ileus, the second was a control group (no manipulation or any CORM treatment), and the last group was treated with CORM-3 at 3 hours and 1 hour before the surgical procedure. An increase in leukocytes was seen in the intestinal muscularis beginning between 3 and 6 hours in the IM mice. The excess leukocytes were reduced with mice that were exposed to CORM-3 prior to the operation. Moreover, intestinal contractility was observed to be restored in the CORM-treated mice. When tested for expression levels of IL-6, monocyte chemoattractant protein-1, and intercellular adhesion molecule-1, as well as inducible NOS activity, were all seen to be decreased following CORM-3 treatment. In contrast, all these parameters were increased with the non-CORM-treated mice. The IM-treated mice demonstrated higher levels of oxidative stress when compared to the mice treated with CORM-3, which displayed significantly reduced oxidative stress levels. From these results, it was concluded that CORM-3 reversed the damaging effects due to postoperative ileus.

## 11. Effect of CORMs in the Lungs

The beneficial effect of CO on lung diseases is currently under much debate. Although *in vivo* models of lung injury induced by acid aspiration [106], aeroallergens [107], hyperoxia [108], and mechanical stretch [109] indicated efficacy of inhaled CO, and other studies [110, 111] did not prove any protective impact of CO. Abid et al.[74] have performed an experiment that proposed beneficial effects of CO on reverse pulmonary hypertension (PH), a complication that occurs as a result of certain diseases. CORM-3 exposure was seen to prevent PH ventricular hypertrophy and distal pulmonary artery muscularization in hypoxia-induced mice. CORM-3 treatment also reversed PH in smooth muscle promoter 22 serotonin transporter mice by decreasing Ki67. Furthermore, CORM-3 increased p21 mRNA and protein levels in lungs. Taken together, the data suggested that through its modulation of p21, CORM-3 may serve as an effective treatment for PH. However, when CORMs were tested under the influence of hypoxic pulmonary vasoconstriction (HPV) using an *in vitro* model, it was observed that the CORMs did not significantly diminish HPV [75]. Furthermore, CORM administration did not result in the inhibition of CYP, a cytochrome that regulates HPV. When higher concentrations of CORMs were used, irreversible pulmonary vasoconstriction resulted. However, inhaled CO led to a decrease in HPV and CYP. Thus, using CORMs for the purpose of treating hypoxic pulmonary vasoconstriction is not particularly effective. Nevertheless, it is possible that the application of CORMs *in vivo* may lead to different results, and they may even have inhibitory effects on HPV.

## 12. Influence of CORMs on the Ocular System

That CO is a molecule crucial to the ocular system has only recently been noted. Low concentrations of CO can exert helpful effects in various ocular conditions, for example, in glaucoma [112]. Furthermore, Stagni et al. [76] have reported that CORM-3 administration lowered intraocular pressure in rabbits. A considerable decrease of intraocular pressure was noted 30 min after CORM-3 exposure for up to 24 hours. A maximum effect was observed 6 hours after addition of a 1% dose of CORM-3; this produced a maximum drop in pressure of 12 mm Hg. Also, treatment with 0.01% and 0.1% CORM-3 solutions generated IOP drops of 3 and 7 mm Hg, respectively. Treatment with the inactive form, iCORM-3, had no effect on the intraocular pressure, suggesting that the CO released by CORM-3 is responsible for the observed this decrease.

## 13. Cardiovascular Effects of CORMs

Anderson [113] have utilized an electrocardiogram to evaluate myocardial damage due to CO poisoning. Five of six CO-poisoned patients were observed to have considerable electrocardiographic abnormalities. ST-segment and T-wave abnormalities were detected, although atrial fibrillation and intraventricular block were also sometimes seen. The persistence of electrocardiographic abnormalities was variable; in one patient, minor changes were observed and then only for a few hours, whereas, in another, the changes were more severe and persisted for more than 24 days. Some patients had durations of electrocardiographic abnormalities lasting up to 4 months. Myocardial injury and necrosis, focal areas of leukocyte infiltration, and punctate hemorrhages were seen histologically as well. Furthermore, CO exposure of 250 ppm induced endothelium-dependent and -independent vascular relaxation abnormalities in rats; these abnormalities were caused by a decrease in cardiac cGMP/cAMP ratio [114]. The anomalies in coronary vascular relaxation were observed in the presence of an increase in heart contractility and cardiac mitochondrial respiration inhibition. It was concluded that CO promoted deformities in coronary vascular relaxation, myocardial contractility, and mitochondrial respiration and that these may induce heart hypoxia. In another study by Andre et al. [115], it was observed that chronic CO exposure reduced the contraction of single rat ventricular myocyte due to the decrease of both systolic Ca^2+^ release and myofilament Ca^2+^ sensitivity. Furthermore, the velocity of both the onset and the relaxation of contraction was significantly reduced, and a slowing of the Ca^2+^ transient decay was seen. Another finding was that CO in high concentrations could induce cardiac arrhythmia. Since CO had no effect on the QT interval of the PQRST deflection seen in the electrocardiogram, its effect on prolonging the duration of the action potential and increasing diastolic Ca^2+^ concentration all together support the hypothesis that the ventricular arrhythmia observed upon CO exposure is caused by an overload of Ca^2+^.

From these three reports, exposure to CO results in severe cardiac arrhythmia, decreased heart contractility, and heart hypoxia. But when CORMs were utilized, different effects were observed due to their low concentration and controlled targeting in the body. For instance, a recent investigation by Segersvärd et al. has shown CORM-3 (but not iCORM-3) improves cardiac structure and function of rats following experimentally induced myocardial infarction [79]. CORM-2 has also displayed cardioprotective behaviour. Tsai et al. [78] have demonstrated that aortic smooth muscle cell migration induced by angiotensin II is attenuated by CORM-2 through inhibition of matrix metalloproteinases-9 expression and ROS/interleukin-6 generation. In addition, Soni et al. [77] have suggested that CORM-2 treatment may improve the heart's function when it is jeopardized by doxorubicin (DXR), a commonly used antitumor agent for heart cancer. DXR can cause cardiotoxic effects, which can lead to cardiomyopathy and heart failure. The mechanism of DXR-induced cardiotoxicity has been associated with excess ROS formation, mitochondrial and DNA damage, and apoptosis. Chronic treatment with CORM-2 can protect the myocardium from DXR exposure. It was also observed that CORM-2 exerted beneficial cardioprotective effects against DXR-induced cardiotoxicity *in vivo* by means of decreasing oxidative stress and apoptosis. Although CORM-2 may possess this effect, it can only exert it under certain concentrations; there is a thin line between the therapeutic benefits and toxic effects of CORM-2, which suggests that it has a narrow therapeutic index. However, there was no increase in the amount of CO-Hb at higher concentrations of CORM-2, indicating that the toxicity at higher doses of CORM-2 may be due to some mechanism other than the increased CO-Hb. Moreover, decreased amounts of serum creatine kinase and lactate dehydrogenase were found for the CORM-2 + DXR-treated groups when compared to DXR alone. Furthermore, increased antioxidant and decreased malondialdehyde contents were seen for the CORM-2 + DXR-treated hearts when compared to those for DXR alone.

Finally, Abramochkin et al. [116] have evaluated the electrophysiological effects of CORMs on murine myocardium. Two effects were noted: action potential shortening in the working myocardium and an acceleration of the sinus rhythm. Thus, an increase in heart rate was observed after CO exposure; this property may suggest that CORMs could have a potential use as a positive chronotropic drug.

## 14. Procoagulant and Anticoagulant Effects of CORMs

Several studies have suggested that CORMs are procoagulant agents and Nielsen et al. [80] have shown that exposure of human plasma to CORM-2 caused an increased speed of growth and strength of thrombi. After tissue factor activation, both CORM-2 and iCORM-2 treatment resulted in a decrease in the time to onset of coagulation, an increase in the velocity of clot growth and an increase in the clot strength in all three types of plasma. Plasma exposed to CORM-2 had a velocity of clot growth that was more than double that of the unexposed samples; however, the samples treated with iCORM only displayed a 50% increase. After celite activation, CORM-2 exposure increased the velocity of clot growth by 56% and clot strength by 57%, when compared with the data for the untreated plasma samples. With respect to the mechanism of CORM-2 enhancement of coagulation, it appears to be due to the increased thrombin-fibrinogen interactions and to be independent of FXIII activation.

Whether CORM-2 could enhance plasmatic coagulation and decrease bleeding times were assessed further in the work conducted by Nielsen et al. [81] and Machovec et al. [82]. Contrary to the findings in Nielsen et al.'s 2009 article [80], there was no significant effect seen on time to maximum rate of thrombus generation induced by CORM-2 in either study. However, the velocity of thrombus formation and the clot strength both increased with CORM-2 administration, in agreement with the study by Nielsen et al. [80]. In addition, CORM-2 treatment caused a considerable decrease in the bleeding time values. The results from these studies suggest that CORM-2 has the potential to improve hemostasis due to its ability to increase the strength of clots and the velocity of clot formation [80–82]. More recently, Nielsen et al. have demonstrated that CORM-2 (but not iCORM-2) is able to attenuate snake venoms with fibrinogen- or thrombin-like activity [83–86].

In addition to displaying procoagulant effects, CORMs also possess anticoagulant properties. Thrombosis, the local blood clotting condition that leads to the obstruction of blood flow through the circulatory system, is a major complication in many vascular pathological conditions. Chen et al. [87] have shown that CO, a product of the HO-1 reaction, can result in antithrombotic effects when platelet mediated thrombus formation was inhibited in the graft. HO-1-deficient mice were seen to result in a 0% survival rate within 4 days due to the development of arterial thrombosis after aortic transplantation. However, recipients that normally expressed HO-1 demonstrated 100% graft acceptance and survival for more than 56 days. When analyzed histologically, treatment with CORM-2 revealed a reduced platelet aggregation within the graft, whereas the non-CORM-2-treated recipients exhibited an elevated aggregation. Therefore, it appears that CO can indeed protect against vascular arterial thrombosis in murine aortic allotransplantation.

Another study that has illustrated the anticoagulant impact of CORMs is that performed by Kramkowski et al. in which they demonstrated that CORM-3 and CORM-A1 inhibit platelet aggregation *in vivo* [88]. When both CORMs were administered intravenously at micromolar concentrations, there was an inhibition of arterial thrombus formation. The effect of CORM-A1 is dependent on the concentration, and this compound is more potent than CORM-3. In comparison to CORM-A1, CORM-3 only inhibited thrombus formation the best at the highest concentration (30 *μ*mol/kg). A considerable decrease in thrombus weight was correlated with the inhibition of platelet aggregation only for the CORM-A1-treated group, perhaps due to its constant and gradual mechanism of CO release. Neither CORM-A1 nor CORM-3 had any effect on the plasma concentration of active tissue plasminogen activator. CORM-3, but not CORM-A1, was noted to lead to a decrease in the concentration of fibrinogen, fibrin generation, and prolonged prothrombin time. Although both CORMs decreased platelet accumulation in thrombus, it was only CORM-A1 that could inhibit platelet activation to phosphatidylserine on their surface.

## 15. CORM Toxicity

The toxicity of CO is thought to primarily arise owing to its preferential binding to Hb. This is proposed to reduce oxygen delivery to tissues. In general, though, any five-coordinate ferrous heme can bind CO, and thus its toxicity will be a result of the combination of these interactions. Indeed, almost all ferrous metalloproteins are potential substrates for CO, and thus the number of metabolic pathways to be considered in these questions of toxicity is enormous. However, CO in low physiological doses is considered safe, with endogenous CO production (arising from heme catabolism) producing CO-Hb levels of about 1% [117, 118]. In fact, low-dose exposure via inhalation has been reported as therapeutic for several conditions [119], although the effects of chronic low-dose exposure are still unclear [118]. High-dose CO exposure, on the other hand, is well established as being toxic. Any CO-Hb levels in the range 15–20% are associated with mild symptoms, including headache and nausea, but more severe effects can be induced at levels >20% and death can occur when levels are greater than 60% [118, 120]. As is common with other gaseous metabolic products such as NO or H_2_O, the excretion pathway is via gas exchange in the lung. From this perspective, CO-Hb is an excretion transporter.

Owing to the potential toxicity of CO, CORMs have been proposed to provide increased CO concentrations for therapeutic purposes without generating toxic CO-Hb levels, which would otherwise be produced through inhalation. This approach has largely been achieved, for example, with CORM-2 [121] and CORM-3 [122], which produce CO-Hb levels lower than the acceptable limits set by the FDA (12–14%) [123]. There is a concern, however, regarding the cytotoxicity of CORMs themselves, particularly of the CO-carrying scaffolds that persist after CO release. Indeed, Seixas et al. have shown that despite the anti-inflammatory activity of CORM-2 and CORM-3 in reducing NO production, these CORMs induced hemagglutination and were hemolytic [124]. CORM-2 has also been associated with disturbing vitamin D3 metabolism in mice [125]. It can produce excessive eryptosis in erythrocytes, which may lead to pathological conditions such as anemia [126] and it has shown significant cellular toxicity by reducing cell viability, decreasing mitochondrial enzyme activity, and causing necrosis in cardiomyocytes and kidney cells [127]. Furthermore, there appears to be rather narrow therapeutic windows for CORM-2 with regard to cardioprotection [77] and for CORM-2 and -3 in treating oxidative stress and ROS production (as described above) [64]. Of perhaps more concern are the effects of consecutive CORM dosing. CORM-3 and other ruthenium-based CORMs as well as a variety of chromium-, molybdenum-, and tungsten-containing CORMs have been shown to cause severe liver and kidney damage in rats after consecutive exposure [128] and, as mentioned above, long-term exposure of CORM-2 in mice following kidney transplant caused lymphocyte accumulation and atrophy of the glomerulus [67].

A frequent test for CORM toxicity and for their CO release physiology is the use of their expected decarbonylation products (iCORMs). It is important to remember though that these derived complexes will have metals in lower oxidation states and thus potentially are able to bind and perhaps activate O_2_. In addition, many of these centers will have accessible redox chemistry and the potential for their interfering with electron transfer pathways in cell membranes or even the cytosol is thus an important consideration.

It is thus clear that CORM cytotoxicity is an essential property to consider when developing these types of compounds for therapeutic purposes and it is an important growing area of study. Motterlini et al. have shown that increasing the water solubility and controlling the rate of CO release of several iron-containing CORMs (CORM-307, -308, -314, and -319) can assist in limiting potential harmful side-effects [129]. Development of compounds that release CO in specific tissue is another approach being considered to reduce unwanted toxicity. Such compounds include PhotoCORMs which release CO only when irradiated with light [13]; enzyme triggered-CORMs which are activated intracellularly, for example, by esterase activity [130, 131]; and CORMs covalently attached to carriers such as nanoparticles [132], micelles [133], or dendrimers [134]. Others have opted to circumvent possible metal-based toxicity by developing transition metal-free CORMs such as CORM-A1 [135]. The continued work in this area and rational design of CORMs, particularly with careful consideration of the “drug sphere” of these molecules, are vital to the development of safe and therapeutically useful CORMs.

## 16. Summary and Future Outlook

This overview of the recent literature on CORMs illustrates their potential benefits for treating a wide array of diseases and conditions (Table 1), but also highlights some important disadvantages and limitations that need be addressed in their continued development as possible therapeutics. Further research into the *drug sphere* proposed by Romão et al. [6] will certainly aid this goal. An additional consideration for the future development of CORMs as therapeutic agents is obtaining better mechanistic understanding of the modes of action for these compounds under physiological and pathological conditions. While *in vitro* measurements of CO release have been routinely investigated spectrophotometrically, using deoxymyoglobin, they can be somewhat unreliable [22] and are not necessarily representative of the biochemistry that occurs within a cellular environment. Information obtained via direct in situ measurement of the rates and amounts of CO released within cells are of greater value. Recently developed CO sensors [27] are proving promising in this regard, as are fluorescent/luminescent CORM derivatives, which provide a means to track CORMs and monitor their CO release [136–138]. Continued studies on the metabolism and retention/clearance of CORMs, as well as the cytotoxicity of these molecules and their by-products, are also urgently needed.

One question that needs to be addressed is whether CO or its carrier scaffolds are responsible for the observed effects, be they beneficial or detrimental. Several investigations have made use of inactivated CORMs as controls; however, it remains to be seen if these iCORMs are equivalent to the by-products generated in situ following CO release or if they are metabolized in the same manner. It has not yet been established whether the effect due to a CORM is due to released CO gas, the CORM itself or any of the intermediates, or final breakdown products [139]. An important proviso is that even the most direct release of CO will generate a 16-electron coordinatively unsaturated intermediate that can engage in a wide range of organometallic chemistry in situ. There is no necessary connection between these reactive intermediates and their ultimate decomposition products made ex vivo. A credible attempt to address the problem of the unknown structures of the iCORM intermediates has been recently been reported by the Schatzschneider group [140]. As mentioned earlier, the photoactivable complex, [Mn(CO)_3_(tpa-*κ*^3^*N*)]Br, is a CORM prodrug that is stable in solution in the dark. However, photoactivation at 365 nm leads to CO release transfer to heme proteins, as demonstrated by the standard myoglobin assay. Interestingly, several different iCORM intermediates could be detected by solution IR spectroscopy and assigned using DFT vibrational calculations. Further information regarding the association of CORMs (and iCORMs) with proteins would provide important insights into the mechanism of action or toxicity of these molecules. Such investigations on CORM-3 have already provided evidence in this regard [141]. Limiting the nonspecific interactions of CORMs with proteins and tissues, however, is a strong driving force in the development of new compounds and this will continue to be an important avenue of research.

There is a need for more concrete information pertaining to the identity of specific CO targets, their binding mechanism, and the subsequent effects induced by the next generation of CORMs that lead to the observed therapeutic benefits, such as those described for the variety of conditions above. Many studies have implicated heme-containing proteins (other than Hb and Mb) as targets, despite their generally low affinity for CO [142]. This does not preclude their involvement, however, as molecules that enhance CO binding affinity have been reported for mitochondrial cytochrome c (naturally occurring cardiolipin) [143] and sGC (synthetic compound YC-1) [144]. These findings lead to the question if CORMs (or iCORMs) themselves could be acting as CO-binding enhancers or if there are other, as yet, unidentified biomolecules that augment the CO-binding affinity of proteins. In any event, efforts to further our understanding of the biological behaviour of CORMs will certainly be worthwhile and important in the advancement towards using these compounds in therapeutic applications.

Another area to be examined is the reversibility of CORM interactions. There does not seem to have been much research yet on the effect of hyperoxia on CORM treatment, that is, the exposure of tissue and organs to excess amounts of O_2_. Does the introduction of O_2_ reverse the effect of CO production from CORMs? There has been one study on the exposure of the tricarbonyldichlororuthenium(II) dimer (CORM‐2; ≥5 *μ*M) to erythrocytes for 24 h that significantly increased the formation of carboxyhemoglobin, which could be partially reversed by the introduction of O_2_ [126].

Finally, the latest development in CO delivery to biological systems is the new field of organic CO prodrugs. A summary of this new area has just been published in *Accounts of Chemical Research* in an article entitled “Strategies Toward Organic Carbon Monoxide Prodrugs” [145]. A specific example of this topic from the same research group at Georgia State University is their study of organic CO prodrugs activated by endogenous ROS [146]. The absence of a metal center from the delivery system may well prove to be an important factor in future clinical studies in terms of avoiding the potential toxicity of the organometallic CORMs. Two types of CO prodrug have been developed, which are precursors to norbornadien-7, ones that release CO under mild conditions [145].

In conclusion, despite all the research activity focussing on the possibility of controlling CO delivery in clinical situations, one must be be conscious of the fact that the use of CORMs and now organic CO prodrugs may not always be beneficial as even such limited CO exposure may result in damage of internal organs and possibly have serious effects on the fetuses of pregnant women [147].

## Figures and Tables

**Table 1:** A summary of the potential therapeutic applications of CORMs.

	CORM	Effect	Ref.
*Bacteria:*			
*E. coli*	CORM-2	Decreased viability of uropathogenic isolates and reduced colonization of human bladder epithelial cells	[17]
		Suppressed cell membrane respiration in the EC598 strain	
	PhotoCORM		[18]
	TryptoCORM	Reduces cell viability by > 99.9%	[19]
*N. gonorrhoeae*	TryptoCORM	Reduces cell viability by > 99%	[20]
*H. pylori*	CORM-2	Reduced cell viability via inhibition of Ni-containing urease	[21]
	CORM-3		
*S. typhimurium*	CORM-3	Reduced growth and viability	[22]
*P. aeruginosa*	CORM-2	CORM-2, -3, and -371 reduced bacterial O_2_ consumption and displayed bactericidal properties	[23]
	CORM-3		
	CORM-371	CORM-A1 slowed bacterial growth (bacteriostatic)	
	CORM-A1		
	[MnBr_2_(CO)_4_]	Varied reduction of cellular growth for a variety of strains	[24]
*Neurodifferentiation and neuroprotection:*			
Neurodifferentiation	CORM-A1	Improved neuronal differentiation and yield in NT2 cell line by promoting oxidative metabolism	[25]
Neuroprotection	CORM-2	Increased viability of neural stem cells and reduced number of apoptotic cells	[26]
		Lessened mitochondrial damage and improved neurological function of mice after induced cardiac arrest	[27]
	ALF186	Prevented apoptosis in nerve cells simulating ischemic respiratory arrest by increasing cellular cGMP levels	[28]
	ALF492	Protected mice against cerebral malaria	[29]
Cochlear inflammation	CORM-2	Inhibited MCP-1/CCL2 upregulation, reducing oxidative stress and protecting against cochlear inflammation	[30]
Neuroinflammation	CORM-3	Reduced inflammatory response in BV-2 microglial cells by reducing NO production	[31]
		Suppresses interleukin-1*β*-induced inflammatory responses	[32]
*Nociception and diabetes:*			
Neuropathic pain	CORM-2	Attenuated mechanical allodynia, thermal hyperalgesia, and thermal allodynia when used in combination with the antinociceptive JWH-015	[33]
		Reduced sciatic nerve injury-induced mechanical and thermal hypersensitivity by attenuating spinal microglial activation and expression of NOS1/2 and CD11b/c proteins	[34]
Diabetes	CORM-A1	Facilitated beta cell regeneration by reducing T-helper cell counts and TGF-*β* and Ki-67 expression	[35]
*Inflammatory disease:*			
Colitis	CORM-2	Reduced cell survival of colitis-inducing cells	[36]
	CO-HbV	Reduced tissue damage and prolonged survival of mice with induced colitis	[37]
Bacterial LPS-induced inflammation	CORM-2	Prevented LPS-mediated inflammation by reducing TLR4/MD2 expression on dendritic cell surfaces and protected mice against increased neutrophil counts associated with septic inflammation	[38]
Tumour necrosis factor *α*-induced inflammation	CORM-2	Induced p65 glutathionylation which protects cysteinyl residues from irreversible oxidation	[39]
Inflammatory disease (cont.):			
Inflammation-induced blood clotting	CORM-2	Decreased blood clotting in human umbilical vein endothelial cells by suppressing MAPK and NF-*κ*B signaling pathways	[40]
Uveitis	CORM-A1	Improved retina morphology and expression of IFNgamma and IL-17A was lowered and IL-10 raised in uveitis-induced mice	[41]
Chronic inflammatory pain	CORM-2	Reduced mechanical allodynia and thermal hyperalgesia in mice and diminished upregulation of NOS1 expression	[42]
Intestinal barrier function	CORM-2	Improved barrier function of intestinal epithelial cells by suppressing phosphorylation of the myosin light chain	[43]
Periodontal disease	CORM-3	Inhibited nuclear translocation of NF-*κ*B and reduced DNA binding of p65/p50 subunits	[44]
Vascular inflammation	CORM-3	Inhibited neutrophilic myeloperoxidase activity	[45]
*Sepsis and associated conditions:*			
Oxidative stress	CORM-2	Reduced oxidative stress during sepsis by increasing HO-1 expression	[46]
NO-induced lipid peroxidation	CORM-2	Attenuated inducible NO synthase and NO production	[46]
CLP-induced sepsis	CORM-2	Improved morphology of intestinal mucosa during sepsis, protecting against LPS-induced intestinal damage	[47]
		Reduced mortality of mice with sepsis-induced acute kidney injury by reducing biomarkers	[48]
Septic lung injury	CORM-3	Restored downregulated annexin A2 levels to normal in LPS-induced lung sepsis	[49]
Myocardial dysfunction	CORM-3	Improved myocardial function in cardiac fibroblasts of septic mice by inhibiting activation of the NLRP3 inflammasome	[50]
Abnormal platelet coagulation	CORM-2	Abnormal platelet activation was reduced by inhibition of glycoprotein-mediated HS1 phosphorylation	[51]
Hyperglycemia	CORM-2	Suppression of hepatic glucose metabolism in mice	[52]
Recruitment of PMN leukocytes	CORM-3	Reduced leukocyte infiltration and attenuated several (but not all) proteins expressed during sepsis	[53]
*Obesity:*			
Dietary-induced	CORM-A1	Reduced weight gain, aided weight loss, and increased lean body mass in mice receiving a high-fat diet	[54]
	CORM-2	Reduced leptin resistance and led to lower body weight of animals fed high-fat diet	[55]
Hyperglycemia	CORM-A1	Decreased hyperglycemia and reduced plasma insulin levels	[54]
*Angiogenesis, aggregation, and cancer:*			
Angiogenesis	CORM-2	Prevented endothelial cell migration and proliferation induced by vascular endothelial growth factor and suppressed phosphorylation of retinoblastoma protein, halting extreme cell replication	[56]
Cancer	CORM-401	Promoted vasorelaxation of precontracted aortic rings	[57]
	CORM-2	Increased survival of mice with A20 lymphoma tumours when encapsulated by folic acid-tagged protein nanoemulsions	[58]
		Prevented global protein synthesis in pancreatic stellate cells	[59]
	PhotoCORM	Reduced cell biomass upon irradiation at 365 nm	[60]
Cell aggregation	CORM-2	Decreased binding affinity of a integrin-specific ligand and lead to reduced cellular aggregation	[61]
*Hemorrhagic shock and postresuscitation injuries:*			
Hemorrhagic shock	CORM-A1	Maintained levels of fenestrations, cells, and adherent leukocytes by reducing expression of cytokines	[62]
	CORM-3	Increased frequency of live human umbilical vein endothelial cells; reduced apoptosis and decreased mitochondrial transmembrane potential and reduced tissue necrosis	[63]
Postresuscitation myocardial injury	CORM-2	Reduced myocytolysis and damage from myocardial fibers and decreased cardiac mitochondrial ROS	[64]
Hypoxia reoxygenation	CORM-3	Conserved cell viability	[65]
Cardiac transplantation	CORM-3	Prolonged survival of rats after heart transplantation	[65]
		Improved coronary flow in mice following heart transplantation	[66]
Kidney transplantation	CORM-2	Pretreating donor rats improved renal histology and function in recipients and long term treatment, however, produced excess lymphocyte accumulation and glomerulus atrophy	[67]
	CORM-3		
*Gastric, intestinal, kidney, and liver disorders:*			
Gastric disorder	CORM-2	Reduced formation of mucosal lesions caused by alendronate (osteoclast inhibitor) in rats stressed by water immersion	[68]
Liver injury	CORM-A1	Reduced hepatocyte cell death by decreasing CK18 cleavage products and lowering RIP3 expression	[69]
Hepatitis	CORM-A1	Significantly reduced deaths in a murine model of autoimmune hepatitis	[70]
Nephrotoxicity	CORM-3	Reduced cell damage induced by cisplatin in renal epithelial cells by suppressing caspase-3 activity and prevented apoptosis and kidney mass loss	[71]
Renoprotection	CORM-3	Increased viability of normal and cancerous human renal cells that were subjected to cisplatin-induced toxicity and ischemia-reperfusion injury	[72]
Intestinal disorder	CORM-3	Partially restored intestinal contractility in mice presenting postoperative ileus and reduced oxidative stress levels	[73]
*Lungs:*			
Pulmonary hypertension	CORM-3	Prevented ventricular hypertrophy and distal pulmonary artery muscularization in hypoxia-induced mice	[74]
		Resulted in irreversible pulmonary vasoconstriction in an in vitro hypoxic pulmonary vasoconstriction model	[75]
*Ocular system:*			
Intraocular pressure	CORM-3	Lowered intraocular pressure in rabbits	[76]
*Cardiovascular effects:*			
Cardioprotection/toxicity	CORM-2	Decreased oxidative stress and apoptosis induced by DXR (antitumor agent) in a narrow therapeutic window	[77]
		Attenuated angiotensin II-induced aortic smooth muscle cell migration by inhibiting matrix metalloproteinase-9 expression and ROS/interleukin-6 generation	[78]
	CORM-3	Improved recovery of cardiac structure and function following myocardial infarction in rats	[79]
*Pro- and anticoagulant effects:*			
Procoagulation	CORM-2	Increased strength and velocity of clot formation	[80–82]
		Attenuates snake venom with fibrinogenolytic and thrombin-like activity	[83–86]
Anticoagulation	CORM-2	Reduced platelet aggregation in aortic allograft recipient mice	[87]
	CORM-3	Decreased arterial thrombus formation	[88]
	CORM-A1		

## Abbreviations

AKI: Acute kidney injury
IECs: Intestinal epithelial cells
ALT: Alanine transaminase
IL: Interleukin
AST: Aspartate transaminase
LPS: Lipopolysaccharide
CB1R/CB2R: Cannabinoid receptor 1/2
LTP: Long-term potentiation
CLP: Cecal ligation and puncture
Mb: Myoglobin
CO: Carbon monoxide
MD2: Myeloid differentiation factor-2
CO-Hb: Carboxyhemoglobin
MPO: Myeloperoxidase
CO-HbV: CO-bound hemoglobin encapsulated liposomes
NO: Nitric oxide
NOS1/NOS2: Nitric oxide synthase 1/2
CORM: CO-releasing molecule
NSC: Neural stem cell
CP: Cisplatin
PH: Pulmonary hypertension
DCs: Dendritic cells
PMN: Polymorphonuclear leukocytes
DSS: Dextran sulphate sodium
PSCs: Pancreatic stellate cells
DXR: Doxorubicin
Rb: Retinoblastoma protein
ER: Endoplasmic reticulum
RBC: Red blood cells
ESBL: Extended-spectrum beta-lactamase
ROS: Reactive oxygen species
ROSC: Return of spontaneous circulation
Hb: Hemoglobin
sGC: soluble guanylate cyclase
HO: Heme oxygenase
STZ: Streptozotocin
HPV: Hypoxic pulmonary vasoconstriction
TLR4: Toll-like receptor 4
UPEC: Uropathogenic isolates of *E. coli*
HS/R: Hemorrhagic shock and resuscitation
UTI: Urinary tract infections
VEGF: Vascular endothelial growth factor
HUVECs: Human umbilical vein endothelial cells
WRS: Stress by water immersion and restraint in cold water
iCORM: inactivated CORM.

## References

[1] Motterlini R. (2002). Carbon monoxide-releasing molecules: characterization of biochemical and vascular activities. *Circulation Research*.

[2] Motterlini R., Haas B., Foresti R. (2012). Emerging concepts on the anti-inflammatory actions of carbon monoxide-releasing molecules (CO-RMs). *Medical Gas Research*.

[3] Heinemann S. H., Hoshi T., Westerhausen M., Schiller A. (2014). Carbon monoxide–physiology, detection and controlled release. *Chemical Communications*.

[4] Murray T. S., Okegbe C., Gao Y. (2012). The carbon monoxide releasing molecule CORM-2 attenuates *Pseudomonas aeruginosa* biofilm formation. *PLoS One*.

[5] Adamson A. W. (1963). *Advanced Inorganic Chemistry*.

[6] Romão C. C., Blättler W. A., Seixas J. D., Bernardes G. J. L. (2012). Developing drug molecules for therapy with carbon monoxide. *Chemical Society Reviews*.

[7] Daniel J., Seixas S. (2011). Development of CO-releasing molecules for the treatment of inflammatory diseases alfama development of CO-releasing molecules for the treatment of inflammatory diseases.

[8] Abel E. W., Butler I. S., Reid J. G. (1963). 382. The anionic halogenopentacarbonyls of chromium, molybdenum, and tungsten. *Journal of the Chemical Society*.

[9] Kautz A. C., Kunz P. C., Janiak C. (2016). CO-releasing molecule (CORM) conjugate systems. *Dalton Transactions*.

[10] Wright M. A., Wright J. A. (2016). PhotoCORMs: CO release moves into the visible. *Dalton Transactions*.

[11] Schatzschneider U. (2015). Novel lead structures and activation mechanisms for CO-releasing molecules (CORMs). *British Journal of Pharmacology*.

[12] García-Gallego S., Bernardes G. J. L. (2014). Carbon-monoxide-releasing molecules for the delivery of therapeutic co in vivo. *Angewandte Chemie International Edition*.

[13] Gonzales M. A., Mascharak P. K. (2014). Photoactive metal carbonyl complexes as potential agents for targeted CO delivery. *Journal of Inorganic Biochemistry*.

[14] Steiger C., Hermann C., Meinel L. (2017). Localized delivery of carbon monoxide. *European Journal of Pharmaceutics and Biopharmaceutics*.

[15] Ward J. S. (2016). Carbon monoxide-releasing molecules: therapeutic molecules with a wide variety of medical applications. *Organometallic Chemistry*.

[16] Ling K., Men F., Wang W. C., Zhou Y. Q., Zhang H. W., Ye D. W. (2017). Carbon monoxide and its controlled release—therapeutic application, detection and development of carbon monoxide-releasing molecules (CO-RMs). *Journal of Medicinal Chemistry*.

[17] Bang C. S., Kruse R., Johansson K., Persson K. (2016). Carbon monoxide releasing molecule-2 (CORM-2) inhibits growth of multidrug-resistant uropathogenic *Escherichia coli* in biofilm and following host cell colonization. *BMC Microbiology*.

[18] Tinajero-Trejo M., Rana N., Nagel C. (2016). Antimicrobial activity of the manganese photoactivated carbon monoxide-releasing molecule [Mn(CO)_3_(tpa-*κ*^3^*N*)]^+^ against a pathogenic *Escherichia coli* that causes urinary infections. *Antioxidants and Redox Signaling*.

[19] Ward J. S., Lynam J. M., Moir J., Fairlamb I. J. S. (2014). Visible-light-induced CO release from a therapeutically viable tryptophan-derived manganese(I) carbonyl (TryptoCORM) exhibiting potent inhibition against *E. coli*. *Chemistry-A European Journal*.

[20] Ward J. S., Morgan R., Lynam J. M., Fairlamb I. J. S., Moir J. W. B. (2017). Toxicity of tryptophan manganese(i) carbonyl (Trypto-CORM), against *Neisseria gonorrhoeae*. *Medicinal Chemistry Communication*.

[21] Tavares A. F., Parente M. R., Justino M. C., Oleastro M., Nobre L. S., Saraiva L. M. (2013). The bactericidal activity of carbon monoxide-releasing molecules against helicobacter pylori. *PLoS One*.

[22] Rana N., McLean S., Mann B. E., Poole R. K. (2014). Interaction of the carbon monoxide-releasing molecule Ru(CO)3Cl(glycinate) (CORM-3) with *Salmonella enterica* serovar Typhimurium: in situ measurements of carbon monoxide binding by integrating cavity dual-beam spectrophotometry. *Microbiology*.

[23] Desmard M., Foresti R., Morin D. (2012). Differential antibacterial activity against *Pseudomonas aeruginosa* by carbon monoxide-releasing molecules. *Antioxidants and Redox Signaling*.

[24] Flanagan L., Steen R. R., Saxby K. (2018). The antimicrobial activity of a carbon monoxide releasing molecule (EBOR-CORM-1) is shaped by intraspecific variation within Pseudomonas aeruginosa populations. *Frontiers in Microbiology*.

[25] Almeida A. S., Sonnewald U., Alves P. M., Vieira H. L. A. (2016). Carbon monoxide improves neuronal differentiation and yield by increasing the functioning and number of mitochondria. *Journal of Neurochemistry*.

[26] Xie Z., Han P., Cui Z. (2016). Pretreatment of mouse neural stem cells with carbon monoxide-releasing molecule-2 interferes with NF-*κ*B p65 signaling and suppresses iron overload-induced apoptosis. *Cellular and Molecular Neurobiology*.

[27] Wang P., Yao L., Zhou L. L. (2016). Carbon monoxide improves neurologic outcomes by mitochondrial biogenesis after global cerebral ischemia induced by cardiac arrest in rats. *International Journal of Biological Sciences*.

[28] Schallner N., Romão C. C., Biermann J. (2013). Carbon monoxide abrogates ischemic insult to neuronal cells via the soluble guanylate cyclase-cGMP pathway. *PLoS One*.

[29] Pena A. C., Penacho N., Mancio-Silva L. (2012). A novel carbon monoxide-releasing molecule fully protects mice from severe malaria. *Antimicrobial Agents and Chemotherapy*.

[30] Woo J. I., Kil S. H., Oh S. (2015). IL-10/HMOX1 signaling modulates cochlear inflammation via negative regulation of MCP-1/CCL2 expression in cochlear fibrocytes. *Journal of Immunology*.

[31] Bani-Hani M. G. (2006). Modulation of thrombin-induced neuroinflammation in BV-2 microglia by carbon monoxide-releasing molecule 3. *Journal of Pharmacology and Experimental Therapeutics*.

[32] Lin C. C., Yang C. C., Hsiao L. D., Chen S. Y., Yang C. M. (2017). Heme oxygenase-1 induction by carbon monoxide releasing molecule-3 suppresses interleukin-1*β*-mediated neuroinflammation. *Frontiers in Molecular Neuroscience*.

[33] Castany S., Carcolé M., Leánez S., Pol O. (2016). The role of carbon monoxide on the anti-nociceptive effects and expression of cannabinoid 2 receptors during painful diabetic neuropathy in mice. *Psychopharmacology*.

[34] Hervera A., Leánez S., Motterlini R., Pol O. (2013). Treatment with carbon monoxide-releasing molecules and an HO-1 inducer enhances the effects and expression of μ-opioid receptors during neuropathic pain. *Anesthesiology*.

[35] Nikolic I., Saksida T., Vujicic M., Stojanovic I., Stosic-Grujicic S. (2015). Anti-diabetic actions of carbon monoxide-releasing molecule (CORM)-A1: immunomodulation and regeneration of islet beta cells. *Immunology Letters*.

[36] Steiger C., Uchiyama K., Takagi T. (2016). Prevention of colitis by controlled oral drug delivery of carbon monoxide. *Journal of Controlled Release*.

[37] Nagao S., Taguchi K., Miyazaki Y. (2016). Evaluation of a new type of nano-sized carbon monoxide donor on treating mice with experimentally induced colitis. *Journal of Controlled Release*.

[38] Riquelme S. A., Bueno S. M., Kalergis A. M. (2015). Carbon monoxide down-modulates toll-like receptor 4/MD2 expression on innate immune cells and reduces endotoxic shock susceptibility. *Immunology*.

[39] Yeh P. Y., Li C. Y., Hsieh C. W., Yang Y. C., Yang P. M., Wung B. S. (2014). CO-releasing molecules and increased heme oxygenase-1 induce protein S-glutathionylation to modulate NF-*κ*B activity in endothelial cells. *Free Radical Biology and Medicine*.

[40] Maruyama K., Morishita E., Yuno T. (2012). Carbon monoxide (CO)-releasing molecule-derived CO regulates tissue factor and plasminogen activator inhibitor type 1 in human endothelial cells. *Thrombosis Research*.

[41] Fagone P., Mangano K., Mammana S. (2015). Carbon monoxide-releasing molecule-A1 (CORM-A1) improves clinical signs of experimental autoimmune uveoretinitis (EAU) in rats. *Clinical Immunology*.

[42] Negrete R., Hervera A., Leánez S., Pol O. (2014). Treatment with a carbon monoxide-releasing molecule inhibits chronic inflammatory pain in mice: nitric oxide contribution. *Psychopharmacology*.

[43] Mu X., Pan C., Zheng S. (2014). Protective effects of carbon monoxide-releasing molecule-2 on the barrier function of intestinal epithelial cells. *PLoS One*.

[44] Choi E. Y., Choe S. H., Hyeon J. Y., Choi J., Choi I. S., Kim S. J. (2015). Carbon monoxide-releasing molecule-3 suppresses *Prevotella intermedia* lipopolysaccharide-induced production of nitric oxide and interleukin-1*β* in murine macrophages. *European Journal of Pharmacology*.

[45] Patterson E. K., Fraser D. D., Capretta A., Potter R. F., Cepinskas G. (2014). Carbon monoxide-releasing molecule 3 inhibits myeloperoxidase (MPO) and protects against MPO-induced vascular endothelial cell activation/dysfunction. *Free Radical Biology and Medicine*.

[46] Sun B., Zou X., Chen Y., Zhang P., Shi G. (2008). Preconditioning of carbon monoxide releasing molecule-derived CO attenuates LPS-induced activation of HUVEC. *International Journal of Biological Sciences*.

[47] Zhang S., Zheng S., Wang X. (2015). Carbon monoxide-releasing molecule-2 reduces intestinal epithelial tight-junction damage and mortality in septic rats. *PLoS One*.

[48] Lee S., Lee S. J., Coronata A. A. (2014). Carbon monoxide confers protection in sepsis by enhancing beclin 1-dependent autophagy and phagocytosis. *Antioxidants and Redox Signaling*.

[49] Unuma K., Aki T., Noritake K., Funakoshi T., Uemura K. (2017). A CO-releasing molecule prevents annexin A2 down-regulation and associated disorders in LPS-administered rat lung. *Biochemical and Biophysical Research Communications*.

[50] Zhang W., Tao A., Lan T. (2017). Carbon monoxide releasing molecule-3 improves myocardial function in mice with sepsis by inhibiting NLRP3 inflammasome activation in cardiac fibroblasts. *Basic Research in Cardiology*.

[51] Liu D., Liang F., Wang X., Cao J., Qin W., Sun B. (2013). Suppressive effect of CORM-2 on LPS-induced platelet activation by glycoprotein mediated HS1 phosphorylation interference. *PLoS One*.

[52] Liang F., Cao J., Qin W. T., Wang X., Qiu X. F., Sun B. W. (2014). Regulatory effect and mechanisms of carbon monoxide-releasing molecule II on hepatic energy metabolism in septic mice. *World Journal of Gastroenterology*.

[53] Serizawa F., Patterson E., Potter R. F., Fraser D. D., Cepinskas G. (2015). Pretreatment of human cerebrovascular endothelial cells with CO-releasing molecule-3 interferes with JNK/AP-1 signaling and suppresses LPS-induced proadhesive phenotype. *Microcirculation*.

[54] Hosick P. A., AlAmodi A. A., Hankins M. W., Stec D. E. (2016). Chronic treatment with a carbon monoxide releasing molecule reverses dietary induced obesity in mice. *Adipocyte*.

[55] Zheng M., Zhang Q., Joe Y. (2013). Carbon monoxide-releasing molecules reverse leptin resistance induced by endoplasmic reticulum stress. *American Journal of Physiology-Endocrinology and Metabolism*.

[56] Ahmad S., Hewett P. W., Fujisawa T. (2015). Carbon monoxide inhibits sprouting angiogenesis and vascular endothelial growth factor receptor-2 phosphorylation. *Thrombosis and Haemostasis*.

[57] Fayad-Kobeissi S., Ratovonantenaina J., Dabiré H. (2016). Vascular and angiogenic activities of CORM-401, an oxidant-sensitive CO-releasing molecule. *Biochemical Pharmacology*.

[58] Loureiro A., Bernardes G. J. L., Shimanovich U. (2015). Folic acid-tagged protein nanoemulsions loaded with CORM-2 enhance the survival of mice bearing subcutaneous A20 lymphoma tumors. *Nanomedicine: Nanotechnology, Biology and Medicine*.

[59] Schwer C. I., Stoll P., Rospert S. (2013). Carbon monoxide releasing molecule-2 CORM-2 represses global protein synthesis by inhibition of eukaryotic elongation factor eEF2. *The International Journal of Biochemistry and Cell Biology*.

[60] Niesel J., Pinto A., Peindy N’Dongo H. W. (2008). Photoinduced CO release, cellular uptake and cytotoxicity of a tris(pyrazolyl)methane (tpm) manganese tricarbonyl complex. *Chemical Communications*.

[61] Chigaev A., Smagley Y., Sklar L. A. (2014). Carbon monoxide down-regulates *α*4*β*1 integrin-specific ligand binding and cell adhesion: a possible mechanism for cell mobilization. *BMC Immunology*.

[62] Nassour I., Kautza B., Rubin M. (2015). Carbon monoxide protects against hemorrhagic shock and resuscitation-induced microcirculatory injury and tissue injury. *Shock*.

[63] Sener A., Tran K. C., Deng J. P. (2013). Carbon monoxide releasing molecules inhibit cell death resulting from renal transplantation related stress. *Journal of Urology*.

[64] Yao L., Wang P., Chen M. (2015). Carbon monoxide-releasing molecules attenuate postresuscitation myocardial injury and protect cardiac mitochondrial function by reducing the production of mitochondrial reactive oxygen species in a rat model of cardiac arrest. *Journal of Cardiovascular Pharmacology and Therapeutics*.

[65] Clark J. E. (2003). Cardioprotective actions by a water-soluble carbon monoxide-releasing molecule. *Circulation Research*.

[66] Musameh M. D., Green C. J., Mann B. E., Fuller B. J., Motterlini R. (2007). Improved myocardial function after cold storage with preservation solution supplemented with a carbon monoxide–releasing molecule (CORM-3). *Journal of Heart and Lung Transplantation*.

[67] Caumartin Y., Stephen J., Deng J. P. (2011). Carbon monoxide-releasing molecules protect against ischemia–reperfusion injury during kidney transplantation. *Kidney International*.

[68] Magierowski M., Magierowska K., Szmyd J. (2016). Hydrogen sulfide and carbon monoxide protect gastric mucosa compromised by mild stress against alendronate injury. *Digestive Diseases and Sciences*.

[69] Bakhautdin B., Das D., Mandal P. (2014). Protective role of HO-1 and carbon monoxide in ethanol-induced hepatocyte cell death and liver injury in mice. *Journal of Hepatology*.

[70] Mangano K., Cavalli E., Mammana S. (2018). Involvement of the Nrf2/HO-1/CO axis and therapeutic intervention with the CO-releasing molecule CORM-A1, in a murine model of autoimmune hepatitis. *Journal of Cellular Physiology*.

[71] Tayem Y., Johnson T. R., Mann B. E., Green C. J., Motterlini R. (2006). Protection against cisplatin-induced nephrotoxicity by a carbon monoxide-releasing molecule. *American Journal of Physiology-Renal Physiology*.

[72] Yoon Y. E., Lee K. S., Lee Y. J., Lee H. H., Han W. K. (2017). Renoprotective effects of carbon monoxide–releasing molecule 3 in ischemia-reperfusion injury and cisplatin-induced toxicity. *Transplantation Proceedings*.

[73] De Backer O., Elinck E., Blanckaert B., Leybaert L., Motterlini R., Lefebvre R. A. (2009). Water-soluble CO-releasing molecules reduce the development of postoperative ileus via modulation of MAPK/HO-1 signalling and reduction of oxidative stress. *Gut*.

[74] Abid S., Houssaini A., Mouraret N. (2014). p21-dependent protective effects of a carbon monoxide-releasing molecule-3 in pulmonary hypertension. *Arteriosclerosis, Thrombosis, and Vascular Biology*.

[75] Pak O., Bakr A. G., Gierhardt M. (2016). Effects of carbon monoxide-releasing molecules on pulmonary vasoreactivity in isolated perfused lungs. *Journal of Applied Physiology*.

[76] Stagni E., Privitera M. G., Bucolo C., Leggio G. M., Motterlini R., Drago F. (2009). A water-soluble carbon monoxide-releasing molecule (CORM-3) lowers intraocular pressure in rabbits. *British Journal of Ophthalmology*.

[77] Soni H., Pandya G., Patel P., Acharya A., Jain M., Mehta A. A. (2011). Beneficial effects of carbon monoxide-releasing molecule-2 (CORM-2) on acute doxorubicin cardiotoxicity in mice: Role of oxidative stress and apoptosis. *Toxicology and Applied Pharmacology*.

[78] Tsai M. H., Lee C. W., Hsu L. F. (2017). CO-releasing molecules CORM2 attenuates angiotensin II-induced human aortic smooth muscle cell migration through inhibition of ROS/IL-6 generation and matrix metalloproteinases-9 expression. *Redox Biology*.

[79] Segersvärd H., Lakkisto P., Hänninen M. (2018). Carbon monoxide releasing molecule improves structural and functional cardiac recovery after myocardial injury. *European Journal of Pharmacology*.

[80] Nielsen V. G., Kirklin J. K., George J. F. (2009). Carbon monoxide releasing molecule-2 increases the velocity of thrombus growth and strength in human plasma. *Blood Coagulation and Fibrinolysis*.

[81] Nielsen V. G., Chawla N., Mangla D. (2011). Carbon monoxide-releasing molecule-2 enhances coagulation in rabbit plasma and decreases bleeding time in clopidogrel/aspirin-treated rabbits. *Blood Coagulation and Fibrinolysis*.

[82] Machovec K. A., Ushakumari D. S., Welsby I. J., Nielsen V. G. (2012). The procoagulant properties of purified fibrinogen concentrate are enhanced by carbon monoxide releasing molecule-2. *Thrombosis Research*.

[83] Nielsen V. G., Bazzell C. M. (2016). Carbon monoxide attenuates the effects of snake venoms containing metalloproteinases with fibrinogenase or thrombin-like activity on plasmatic coagulation. *Medicinal Chemistry Communications*.

[84] Nielsen V. G., Boyer V. L., Matika R. W., Amos Q., Redford D. T. (2016). Iron and carbon monoxide attenuate Crotalus atrox venom-enhanced tissue-type plasminogen activator-initiated fibrinolysis. *Blood Coagulation and Fibrinolysis*.

[85] Nielsen V. G., Bazzell C. M. (2017). Carbon monoxide releasing molecule-2 inhibition of snake venom thrombin-like activity: novel biochemical “brake”?. *Journal of Thrombosis and Thrombolysis*.

[86] Nielsen V. G. (2018). Crotalus atrox venom exposed to carbon monoxide has decreased fibrinogenolytic activity in vivo in rabbits. *Basic and Clinical Pharmacology and Toxicology*.

[87] Chen B., Guo L., Fan C. (2009). Carbon monoxide rescues heme oxygenase-1-deficient mice from arterial thrombosis in allogeneic aortic transplantation. *American Journal of Pathology*.

[88] Kramkowski K., Leszczynska A., Mogielnicki A. (2012). Antithrombotic properties of water-soluble carbon monoxide-releasing molecules. *Arteriosclerosis, Thrombosis, and Vascular Biology*.

[89] Klinger-Strobel M., Gläser S., Makarewicz O. (2016). Bactericidal effect of a photoresponsive carbon monoxide-releasing nonwoven against *Staphylococcus aureus* biofilms. *Antimicrobial Agents and Chemotherapy*.

[90] Wilson J. L., Wareham L. K., McLean S. (2015). CO-releasing molecules have nonheme targets in bacteria: transcriptomic, mathematical modeling and biochemical analyses of CORM-3 [Ru(CO)3Cl(glycinate)] actions on a heme-deficient mutant of *Escherichia coli*. *Antioxidants and Redox Signaling*.

[91] Verma A., Hirsch D., Glatt C., Ronnett G., Snyder S. (1993). Carbon monoxide: a putative neural messenger. *Science*.

[92] Battish R., Cao G. Y., Lynn R. B., Chakder S., Rattan S. (1998). Heme-oxygenase 2 (HO-2) distribution in anorectum of opossum: colocalization with neuronal nitric oxide synthase (nNOS) and vasoactive intestinal polypeptide (VIP). *Gastroenterology*.

[93] Xue L., Farrugia G., Miller S. M., Ferris C. D., Snyder S. H., Szurszewski J. H. (2000). Carbon monoxide and nitric oxide as coneurotransmitters in the enteric nervous system: evidence from genomic deletion of biosynthetic enzymes. *Proceedings of the National Academy of Sciences*.

[94] Henke K., Kroll N. E., Behniea H. (1999). Memory lost and regained following bilateral hippocampal damage. *Journal of Cognitive Neuroscience*.

[95] Mancuso C., Ragazzoni E., Tringali G. (1999). Inhibition of heme oxygenase in the central nervous system potentiates endotoxin-induced vasopressin release in the rat. *Journal of Neuroimmunology*.

[96] Calingasan N. Y., Chun W. J., Park L. C. H., Uchida K., Gibson G. E. (1999). Oxidative stress is associated with region-specific neuronal death during thiamine deficiency. *Journal of Neuropathology and Experimental Neurology*.

[97] Choi I. S., Cheon H. Y. (1999). Delayed movement disorders after carbon monoxide poisoning. *European Neurology*.

[98] Schipper H. M., Liberman A., Stopa E. G. (1998). Neural heme oxygenase-1 expression in idiopathic Parkinson’s disease. *Experimental Neurology*.

[99] Piantadosi C. A., Zhang J., Levin E. D., Folz R. J., Schmechel D. E. (1997). Apoptosis and delayed neuronal damage after carbon monoxide poisoning in the rat. *Experimental Neurology*.

[100] Durak A. C., Coskun A., Yikilmaz A., Erdogan F., Mavili E., Guven M. (2005). Magnetic resonance imaging findings in chronic carbon monoxide intoxication. *Acta Radiologica*.

[101] Queiroga C. S. F., Alves R. M. A., Conde S. V., Alves P. M., Vieira H. L. A. (2016). Paracrine effect of carbon monoxide–astrocytes promote neuroprotection through purinergic signaling in mice. *Journal of Cell Science*.

[102] Simon T., Pogu S., Tardif V. (2013). Carbon monoxide-treated dendritic cells decrease *β*1-integrin induction on CD8+T cells and protect from type 1 diabetes. *European Journal of Immunology*.

[103] Jung S. S., Moon J. S., Xu J. F. (2015). Carbon monoxide negatively regulates NLRP3 inflammasome activation in macrophages. *American Journal of Physiology-Lung Cellular and Molecular Physiology*.

[104] Bougié A., Harrois A., Duranteau J. (2013). Resuscitative strategies in traumatic hemorrhagic shock. *Annals of Intensive Care*.

[105] Takagi T., Naito Y., Uchiyama K. (2016). Carbon monoxide promotes gastric wound healing in mice via the protein kinase C pathway. *Free Radical Research*.

[106] Nemzek J. A., Fry C., Abatan O. (2008). Low-dose carbon monoxide treatment attenuates early pulmonary neutrophil recruitment after acid aspiration. *American Journal of Physiology-Lung Cellular and Molecular Physiology*.

[107] Chapman J. T., Otterbein L. E., Elias J. A., Choi A. M. K. (2001). Carbon monoxide attenuates aeroallergen-induced inflammation in mice. *American Journal of Physiology-Lung Cellular and Molecular Physiology*.

[108] Otterbein L. E., Otterbein S. L., Ifedigbo E. (2003). MKK3 mitogen-activated protein kinase pathway mediates carbon monoxide-induced protection against oxidant-induced lung injury. *American Journal of Pathology*.

[109] Dolinay T., Szilasi M., Liu M., Choi A. M. K. (2004). Inhaled carbon monoxide confers antiinflammatory effects against ventilator-induced lung injury. *American Journal of Respiratory and Critical Care Medicine*.

[110] Aberg A. M., Abrahamsson P., Johansson G., Haney M., Winso O., Larsson J. E. (2008). Does carbon monoxide treatment alter cytokine levels after endotoxin infusion in pigs? A randomized controlled study. *Journal of Inflammation*.

[111] Mitchell L. A., Channell M. M., Royer C. M., Ryter S. W., Choi A. M. K., McDonald J. D. (2010). Evaluation of inhaled carbon monoxide as an anti-inflammatory therapy in a nonhuman primate model of lung inflammation. *American Journal of Physiology-Lung Cellular and Molecular Physiology*.

[112] Bucolo C., Drago F. (2011). Carbon monoxide and the eye: implications for glaucoma therapy. *Pharmacology and Therapeutics*.

[113] Anderson R. F. (1967). Myocardial toxicity from carbon monoxide poisoning. *Annals of Internal Medicine*.

[114] Favory R., Lancel S., Tissier S., Mathieu D., Decoster B., Nevière R. (2006). Myocardial dysfunction and potential cardiac hypoxia in rats induced by carbon monoxide inhalation. *American Journal of Respiratory and Critical Care Medicine*.

[115] Andre L., Boissière J., Reboul C. (2010). Carbon monoxide pollution promotes cardiac remodeling and ventricular arrhythmia in healthy rats. *American Journal of Respiratory and Critical Care Medicine*.

[116] Abramochkin D. V., Konovalova O. P., Kamkin A., Sitdikova G. F. (2015). Carbon monoxide modulates electrical activity of murine myocardium via cGMP-dependent mechanisms. *Journal of Physiology and Biochemistry*.

[117] Stewart R. D. (1975). The effect of carbon monoxide on humans. *Annual Review of Pharmacology*.

[118] Kao L. W., Nañagas K. A. (2006). Toxicity associated with carbon monoxide. *Clinics in Laboratory Medicine*.

[119] Foresti R., Bani-Hani M. G., Motterlini R. (2008). Use of carbon monoxide as a therapeutic agent: promises and challenges. *Intensive Care Medicine*.

[120] National Research Council (US) Committee on Acute Exposure Guideline Levels (2010). Carbon monoxide acute exposure guideline levels. *Acute Exposure Guideline Levels for Selected Airborne Chemicals: Volume 8*.

[121] Magierowska K., Magierowski M., Hubalewska-Mazgaj M. (2015). Carbon monoxide (CO) released from tricarbonyldichlororuthenium (II) dimer (CORM-2) in gastroprotection against experimental ethanol-induced gastric damage. *PLoS One*.

[122] Wang G., Hamid T., Keith R. J. (2010). Cardioprotective and antiapoptotic effects of heme oxygenase-1 in the failing heart. *Circulation*.

[123] Wegiel B., Hanto D. W., Otterbein L. E. (2013). The social network of carbon monoxide in medicine. *Trends in Molecular Medicine*.

[124] Seixas J. D., Santos M. F. A., Mukhopadhyay A. (2015). A contribution to the rational design of Ru(CO)3Cl2L complexes for in vivo delivery of CO. *Dalton Transactions*.

[125] Feger M., Fajol A., Lebedeva A. (2013). Effect of carbon monoxide donor CORM-2 on vitamin D3 metabolism. *Kidney and Blood Pressure Research*.

[126] Lang E., Qadri S. M., Jilani K., Lupescu A., Schleicheeer E., Lang T. (2012). Carbon monoxide-sensitive apoptotic death of erythrocytes. *Basic and Clinical Pharmacology and Toxicology*.

[127] Winburn I. C., Gunatunga K., McKernan R. D., Walker R. J., Sammut I. A., Harrison J. C. (2012). Cell damage following carbon monoxide releasing molecule exposure: implications for therapeutic applications. *Basic and Clinical Pharmacology and Toxicology*.

[128] Wang P., Liu H., Zhao Q. (2014). Syntheses and evaluation of drug-like properties of CO-releasing molecules containing ruthenium and group 6 metal. *European Journal of Medicinal Chemistry*.

[129] Motterlini R., Sawle P., Hammad J. (2013). Vasorelaxing effects and inhibition of nitric oxide in macrophages by new iron-containing carbon monoxide-releasing molecules (CO-RMs). *Pharmacological Research*.

[130] Romanski S., Kraus B., Schatzschneider U., Neudörfl J. M., Amslinger S., Schmalz H. G. (2011). Acyloxybutadiene iron tricarbonyl complexes as enzyme-triggered CO-releasing molecules (ET-CORMs). *Angewandte Chemie International Edition*.

[131] Mede R., Hoffmann P., Neumann C. (2018). Acetoxymethyl concept for intracellular administration of carbon monoxide with Mn(CO)_3_-based photoCORMs. *Chemistry-A European Journal*.

[132] Dördelmann G., Meinhardt T., Sowik T., Krueger A., Schatzschneider U. (2012). CuAAC click functionalization of azide-modified nanodiamond with a photoactivatable CO-releasing molecule (PhotoCORM) based on [Mn(CO)_3_(tpm)]^+^. *Chemical Communications*.

[133] Hasegawa U., van der Vlies A. J., Simeoni E., Wandrey C., Hubbell J. A. (2010). Carbon monoxide-releasing micelles for immunotherapy. *Journal of the American Chemical Society*.

[134] Govender P., Pai S., Schatzschneider U., Smith G. S. (2013). Next generation PhotoCORMs: polynuclear tricarbonylmanganese(I)-functionalized polypyridyl metallodendrimers. *Inorganic Chemistry*.

[135] Motterlini R., Sawle P., Hammad J. (2005). CORM-A1: a new pharmacologically active carbon monoxide-releasing molecule. *FASEB Journal*.

[136] Pierri A. E., Pallaoro A., Wu G., Ford P. C. (2012). A luminescent and biocompatible PhotoCORM. *Journal of the American Chemical Society*.

[137] Carrington S. J., Chakraborty I., Bernard J. M. L., Mascharak P. K. (2016). A theranostic two-tone luminescent PhotoCORM derived from Re(I) and (2-pyridyl)-benzothiazole: trackable CO delivery to malignant cells. *Inorganic Chemistry*.

[138] Ur G., Axthelm J., Hoffmann P. (2017). Co-registered molecular logic gate with a CO-releasing molecule triggered by light and peroxide. *Journal of the American Chemical Society*.

[139] Gessner G., Sahoo N., Swain S. M. (2013). CO-independent modification of K^+^ channels by tricarbonyldichlororuthenium(II) dimer (CORM-2). *European Journal of Pharmacology*.

[140] Nagel C, McLean, Poole R. K, Braunschweig H., Kramer T., Schatzschneider U. (2014). Introducing [Mn(CO)_3_(tpa-*κ*^3^*N*)]^+^ as a novel photoactivatable CO-releasing molecule with well-defined iCORM intermediates–synthesis, spectroscopy, and antibacterial activity. *Dalton Transactions*.

[141] Santos-Silva T., Mukhopadhyay A., Seixas J. D., Bernardes J. L., Romao C., Romao M. (2011). Towards therapeutic CORMs: understanding the reactivity of CORM-3 with proteins. *Current Medicinal Chemistry*.

[142] Mann B. E., Reedjik J., Poeppelmeier K. (2013). 3.29 signaling molecule delivery (CO). *Comprehensive Inorganic Chemistry (II)*.

[143] Kapetanaki S. M., Slikstoner G., Husu I., Liebl U., Wilson M. T., Vos M. H. (2009). Interaction of carbon monixide with apoptosis—inducing cyytochrome C–cardiolipin complex. *Biochemistry*.

[144] Ibrahim M., Derbyshire E. R., Marletta M. A., Spiro T. G. (2010). Probing soluble guanylate cyclase actgivation by CO and YC-1 usiong resonance Raman spectroscopy. *Biochemistry*.

[145] Ji X., Wang B. (2018). Strategies toward organic carbon monoxide prodrugs. *Accounts of Chemical Research*.

[146] Pan Z., Zhang J., Ji K., Chittavong V., Ji X., Wang W. (2018). Organic CO prodrugs activated by endogenous ROS. *Organic Letters*.

[147] Blackburn S. T. (2007). *Maternal, Fetal, & Neonatal Physiology: A Clinical Perspective*.

